# Analytic results for decays of color singlets to *gg* and $q \bar{q}$ final states at NNLO QCD with the nested soft-collinear subtraction scheme

**DOI:** 10.1140/epjc/s10052-019-7505-x

**Published:** 2019-12-16

**Authors:** Fabrizio Caola, Kirill Melnikov, Raoul Röntsch

**Affiliations:** 1 Rudolf Peierls Centre for Theoretical Physics, Clarendon Laboratory, Parks Road, Oxford, OX1 3PU UK; 2 Wadham College, Oxford, OX1 3PN UK; 3 Institute for Theoretical Particle Physics, KIT, Karlsruhe, Germany; 4 Theoretical Physics Department, CERN, 1211 Geneva 23, Switzerland

## Abstract

We present compact analytic formulas that describe the decay of colorless particles to both $q \bar{q}$ and *gg* final states through next-to-next-to-leading order in perturbative QCD in the context of the nested soft-collinear subtraction scheme. In addition to their relevance for the description of decays like $V \rightarrow q \bar{q}'$, $V= Z,W$, $H \rightarrow b \bar{b}$ and $H \rightarrow gg$, these results provide an important building block for calculating NNLO QCD corrections to arbitrary processes at colliders within the nested soft-collinear subtraction scheme.

## Introduction

The development of an efficient and physically transparent subtraction scheme for next-to-next-to-leading order (NNLO) computations in QCD is an important problem in theoretical particle physics that attracted a lot of attention recently [1–24, 26–30]. However, among the many subtraction schemes that have been proposed, there is not a single one that is generic, fully local and fully analytic (in a sense that all the integrated subtraction terms are available in an analytic form). Given the impressive practical successes of many subtraction schemes in describing physical processes, it is unclear whether or not locality and analyticity are truly essential. However, we believe that it is useful to develop a scheme that is general, physically transparent and efficient, especially in view of the need to extend the functionality of existing subtraction schemes beyond $2 \rightarrow 2$ processes for forthcoming LHC applications.

In Ref. [30], we introduced the nested soft-collinear subtraction scheme. It is based on the idea of sector decomposition [10, 11] but it relies heavily on the phenomenon of color coherence in constructing soft and collinear approximations to matrix elements. This subtraction scheme is local by construction; however, initially, some subtraction terms were not known analytically. Recently, this problem was solved for both the double-soft [31] and triple-collinear [32] subtraction terms so that analytic results for all double-unresolved subtraction terms are now available. Building on that, in Ref. [33] we presented analytic results for the production of a color-singlet final state in hadron collisions obtained within the nested soft-collinear subtraction scheme. In addition to their phenomenological relevance, we view these results as building blocks that should, eventually, allow us to describe arbitrary hard processes at hadron colliders through NNLO QCD. Typically, these building blocks are obtained by partitioning the phase space for a particular process in such a way that only emissions off two hard particles at a time lead to infra-red and collinear singularities when integration over the phase space is attempted. These hard emittors can be both in the initial or in the final state or one of them can be in the initial and the other one in the final state. When looking at the problem of constructing a subtraction scheme from this perspective, the results presented in Ref. [33] should facilitate the description of the two initial-state emittors.

The goal of this paper is to take one further step towards the application of the nested soft-collinear subtraction scheme to the description of generic LHC processes by considering a situation when the hard emittors are in the final state. An important physical example of this situation is decays of colorless particles into a $q \bar{q}$ or *gg* final state. The NNLO QCD results for the $q \bar{q}$ final state have already been used by us in Ref. [34] to describe the decay of the Higgs boson into a massless $b \bar{b}$ pair; however, we did not provide analytic formulas for this final state in that reference. The goal of this paper is to provide such formulas and to supplement them with the analytic results for decays of a color singlet into a *gg* final state.

Although, conceptually, the computation of NNLO QCD corrections to the production and decay of a color singlet are very similar, there are a few differences between the two that are worth pointing out.

- In the case of the double-real corrections to the *gg* final state we need to carefully separate unresolved gluons from the resolved ones. This issue does not appear in case of production where incoming particles are always the hardest ones and their momenta are fixed.
- The computation of the integrated collinear counter-terms requires modifications since, in the initial-state case, the integrated collinear subtraction terms are functions of fractions of the initial energy that a hard parton carries into the hard process, while in case of the final-state emissions one has to integrate over fractions of energies that are shared by partons in the collinear splitting.
- Construction of the double-collinear phase space, i.e. the phase space appropriate for the description of a kinematic situation where singularities occur when each unresolved parton is emitted by a different emittor, is straightforward in the production and non-trivial in the decay cases.
- Obviously, no renormalization of parton distribution functions is needed to describe decay processes; for this reason, cancellation of infra-red and collinear singularities works differently in the production and decay cases.

The rest of the paper is organized as follows. In Sect. 2 we set the stage for the calculations described in the following sections and introduce our notation. We then discuss in detail the calculation of QCD corrections to $H \rightarrow gg$ decay to explain our approach. In particular, in Sect. 3, we present the computation of the NLO QCD corrections to the decay rate $H \rightarrow gg$. In Sect. 4 we discuss how to set up the calculation of NNLO QCD corrections to $H \rightarrow gg$ decay and then consider the $H \rightarrow 4g$ channel in detail. We present our final results for the NNLO QCD corrections to the decay of a color singlet to two gluons in Sect. 4 and to a $q \bar{q}$ final state in Sect. 5. We discuss the validation of our results in Sect. 6, and conclude in Sect. 7. Many useful formulas and intermediate results are collected in several appendices.

## General considerations

We begin by describing common features of QCD corrections to color singlet decays and by introducing notations that we will use throughout the paper. We consider decays of a color-singlet particle *Q* to quarks and gluons. Our goal is to provide formulas that describe NNLO QCD corrections to these decays at a fully-differential level. Specifically, we study the decay process $Q \rightarrow f_i f_j + X$, where $\{f_i,f_j\}$ can be either $\{g,g\}$ or $\{q,{\bar{q}}\}$. We first discuss the decays into the *gg* final state since, compared to $Q \rightarrow q \bar{q}$, the singularity structure of the decay $Q \rightarrow gg$ is more complex. Therefore, once the calculation of the NNLO QCD corrections to $Q \rightarrow gg$ is understood, NNLO QCD corrections to $Q \rightarrow q \bar{q}$ are easily established.

We write the perturbative expansion of the differential decay rate as

2.1 $$\begin{aligned} \mathrm{d}\Gamma = \mathrm{d}\Gamma ^{\mathrm{LO}} + \mathrm{d}\Gamma ^{\mathrm{NLO}} + \mathrm{d}\Gamma ^{\mathrm{NNLO}} + \cdots \end{aligned}$$

The different contributions in Eq. (2.1) are obtained by integrating various matrix elements squared over the phase space of final state particles. To describe this integration in a compact way, we introduce the notation analogous to our earlier papers [30, 33] and define

2.2 $$\begin{aligned}&\left\langle F_\mathrm{LM}(1_{f_1},2_{f_2},\ldots ,n_{f_n}) {\mathcal {O}}(1,\ldots ,n) \right\rangle \nonumber \\&\quad \equiv \frac{\mathcal {N}}{2 m_{H}} \int \prod _{i=1}^{n} [df_{i}] (2\pi )^d \delta ^{(d)}(p_Q - p_1-p_2- \cdots -p_n) \nonumber \\&\qquad \times |\mathcal {M}^\mathrm{tree}|^2(1_{f_1},2_{f_2},\ldots ,n_{f_n}) \mathcal {O}(\{p_1,\ldots ,p_n\}), \end{aligned}$$

where $\mathcal {N}$ is a symmetry factor for identical final-state particles, $d=4-2\epsilon$ is the space-time dimensionality,

2.3 $$\begin{aligned}{}[df_{i}] = \frac{\mathrm{d}^{d-1}p_i}{(2\pi )^{d-1}2 E_i} \theta (E_\mathrm{max}-E_i) \end{aligned}$$

is the phase-space element for a parton $f_i$, $\mathcal {M}^\mathrm{tree}(1_{f_1},\ldots ,n_{f_n})$ is the matrix element for the process

2.4 $$\begin{aligned} Q \rightarrow f_{1}(p_1)+f_2(p_2) + \cdots + f_n(p_n), \end{aligned}$$

and $\mathcal {O}$ is a function that depends on partons’ energies and angles. Furthermore, $E_\mathrm{max}$ is an auxiliary parameter with the dimension of energy that should be large enough to accommodate all events that are allowed by the energy-momentum conservation constraints. Its relevance will become clear in what follows. In the rest of this paper, we will use $E_\mathrm{max}= m_{H}/2$. We note that the explicit constraint on the energy in Eq. (2.3) breaks Lorentz invariance at intermediate stages of the calculation; for this reason all energies in this paper are defined in the rest frame of the decaying particle *Q*.

To describe contributions of loop-corrected processes, we introduce similar quantities^1^

2.5 $$\begin{aligned}&\left\langle F_\mathrm{LV}(1_{f_1},2_{f_2},\ldots ,n_{f_n}) {\mathcal {O}}(1,\ldots ,n) \right\rangle \nonumber \\&\quad \equiv \frac{\mathcal {N}}{2 m_{H}}\int \prod _{i=1}^{n} [df_{i}] (2\pi )^d \delta ^{(d)}(p_Q - p_1-p_2-\cdots -p_n)\nonumber \\&\qquad \times 2{\mathfrak {R}}\big [\mathcal {M}^\mathrm{tree} \mathcal {M}^{\mathrm{1-loop},*}\big ] (1_{f_1},2_{f_2},\ldots ,n_{f_n})\mathcal {O}(\{p_1,\ldots ,p_n\}), \end{aligned}$$

and

2.6 $$\begin{aligned}&\left\langle F_\mathrm{LVV}(1_{f_1},2_{f_2},\ldots ,n_{f_n} {\mathcal {O}}(1,\ldots ,n) )\right\rangle \nonumber \\&\quad \equiv \frac{\mathcal {N}}{2 m_{H}}\int \prod _{i=1}^{n} [df_{i}] (2\pi )^d \delta ^{(d)}(p_Q - p_1-p_2\cdots -p_n)\nonumber \\&\qquad \times \bigg [2{\mathfrak {R}}\big [\mathcal {M}^\mathrm{tree} \mathcal {M}^{\mathrm{2-loop},*}\big ]+\big |\mathcal {M}^\mathrm{1-loop}\big |^2\bigg ]\nonumber \\&\qquad \times (1_{f_1},2_{f_2},\ldots ,n_{f_n})\mathcal {O}(\{p_1,\ldots ,p_n\}). \end{aligned}$$

Finally, we define

2.7 $$\begin{aligned}&\left\langle F_{X}(1,2,\ldots ,n) {\mathcal {O}}(1,\ldots ,n) \right\rangle \nonumber \\&\quad = \sum _{f_1,f_2,\ldots ,f_n} \left\langle F_{X}(1_{f_1},2_{f_2},\ldots ,n_{f_n}) {\mathcal {O}}(1,\ldots ,n) \right\rangle , \end{aligned}$$

where $X=\mathrm{LM,~LV,~LVV}$ and the sum runs over all allowed final states. Using these notations, the three contributions to the differential width Eq. (2.1) are written as

2.8 $$\begin{aligned} \mathrm{d}\Gamma ^\mathrm{LO}= & {} \left\langle F_\mathrm{LM}(1,2)\right\rangle _\delta ,\nonumber \\ \mathrm{d}\Gamma ^\mathrm{NLO}= & {} \left\langle F_\mathrm{LM}(1,2,3)\right\rangle _\delta + \left\langle F_\mathrm{LV}(1,2)\right\rangle _\delta ,\nonumber \\ \mathrm{d}\Gamma ^\mathrm{NNLO}= & {} \left\langle F_\mathrm{LM}(1,2,3,4)\right\rangle _\delta \nonumber \\&+ \left\langle F_\mathrm{LV}(1,2,3)\right\rangle _\delta + \left\langle F_\mathrm{LVV}(1,2)\right\rangle _\delta . \end{aligned}$$

The symbol $\langle \cdots \rangle _\delta$ indicates that the integration over the momenta of partons that are explicitly shown as arguments of a function $F_X$ is not performed, so that the right hand side of Eq. (2.8) provides a fully-differential description of the decay rate.

Starting from next-to-leading order, the individual terms appearing on the right hand sides of Eq. (2.8) are infra-red divergent and cannot be integrated in four dimensions when taken separately. The goal of a subtraction scheme is to rearrange them in the following way

2.9 $$\begin{aligned} \mathrm{d}\Gamma ^{\mathrm{NLO}}= & {} \mathrm{d}\Gamma ^\mathrm{NLO}_{Q\rightarrow 2} + \mathrm{d}\Gamma ^\mathrm{NLO}_{Q \rightarrow 3},\nonumber \\ \mathrm{d}\Gamma ^{\mathrm{NNLO}}= & {} \mathrm{d}\Gamma ^\mathrm{NNLO}_{Q \rightarrow 2} + \mathrm{d}\Gamma ^\mathrm{NNLO}_{Q \rightarrow 3 } +\mathrm{d}\Gamma ^\mathrm{NNLO}_{Q \rightarrow 4}, \end{aligned}$$

where $\mathrm{d}\Gamma ^\mathrm{(N)NLO}_{Q \rightarrow i}$ are finite in four dimensions and contain contributions from final states with at most *i* partons. In Refs. [30, 33] we explained how this can be done for hadroproduction of color-singlet states. We now use a very similar procedure to discuss color singlet decays.

Since the required computations are often quite similar, we do not describe the calculational details if the results for the decay follow easily from the ones for the production. To this end, we note that a detailed introduction to our subtraction scheme can be found in Refs. [30, 33] and we extensively refer to these papers in what follows. In this paper, we highlight differences between the computations required for the production and decay cases and present formulas for color singlet decay to either *gg* or $q \bar{q}$ final states. We begin with the discussion of the NLO QCD corrections to $H \rightarrow gg$.

## Higgs decay to gluons: a NLO computation

We consider the NLO QCD contribution to the differential decay rate of the Higgs boson to two gluons, $H \rightarrow gg$.^2^ We use the notations introduced in the previous section to write

3.1 $$\begin{aligned} \mathrm{d}\Gamma ^{\mathrm{NLO}}= & {} \left\langle F_\mathrm{LM}(1_g,2_g,3_g)\right\rangle _\delta \nonumber \\&+ n_f\left\langle F_\mathrm{LM}(1_q,2_g,3_{\bar{q}})\right\rangle _\delta + \left\langle F_\mathrm{LV}(1_g,2_g)\right\rangle _\delta , \end{aligned}$$

where $n_f$ is the number of massless quarks. We consider the three terms in Eq. (3.1) separately, starting with the real-emission contribution $F_\mathrm{LM}(1_g,2_g,3_g)$. The first step is to identify all possible singularities that may appear in the computation of that contribution and to partition the phase space in such a way that for each partition only a small subset of singularities is present.

An important consequence of any partitioning is the fact that certain partons are identified as “hard”. This means that, for a given partition, we should know exactly which partons cannot produce infra-red singularities. Although there are many ways to construct partitions, we find it convenient to use scalar products of the gluons’ four-momenta $s_{ij} = 2p_i \cdot p_j$ and the energy-momentum conservation

3.2 $$\begin{aligned} p_H^2 = (p_1 + p_2 + p_3)^2 \;\;\; \Rightarrow \;\;\;\; m_{H}^2 = s_{12} + s_{13} + s_{23}, \end{aligned}$$

inside $\left\langle F_\mathrm{LM}(1_g,2_g,3_g)\right\rangle _\delta$. We then write

3.3 $$\begin{aligned}&\left\langle F_\mathrm{LM}(1_g,2_g,3_g)\right\rangle _\delta \nonumber \\&\quad = \left\langle \tilde{s}_{12}F_\mathrm{LM}(1_g,2_g,3_g)\right\rangle _\delta +\left\langle \tilde{s}_{13}F_\mathrm{LM}(1_g,2_g,3_g)\right\rangle _\delta \nonumber \\&\qquad +\left\langle \tilde{s}_{23}F_\mathrm{LM}(1_g,2_g,3_g)\right\rangle _\delta , \end{aligned}$$

where we have introduced the notation $\tilde{s}_{ij} \equiv s_{ij}/m_{H}^2$. We can use the symmetry of the matrix element and the phase space to rewrite this equation as

3.4 $$\begin{aligned} \left\langle F_\mathrm{LM}(1_g,2_g,3_g)\right\rangle _\delta = 3 \left\langle \tilde{s}_{12}F_\mathrm{LM}(1_g,2_g,3_g)\right\rangle _\delta . \end{aligned}$$

Thanks to the prefactor $\tilde{s}_{12}$, gluons $g_1$ and $g_2$ on the right-hand side of Eq. (3.4) must be hard or “resolved” and the only potentially unresolved parton is the gluon $g_3$. This means that the right-hand side of Eq. (3.4) is singular when $g_3$ is soft and when $g_3$ is collinear to either $g_1$ or $g_2$; it is, however, not singular when either $g_1$ or $g_2$ is soft or when $g_1$ and $g_2$ are collinear to each other.

We can follow the approach described in the context of color singlet production [30, 33] to extract singularities from the right hand side of Eq. (3.4). We begin by considering the soft contribution that arises when energy of the gluon $g_3$, $E_3$, becomes small. We find

3.5 $$\begin{aligned}&\lim _{E_3 \rightarrow 0} |{\mathcal {M}}^\mathrm{tree}|^2(1_g,2_g,3_g) \nonumber \\&\quad \approx 2 C_A g_{s,b}^2 \frac{p_1\cdot p_2}{(p_1\cdot p_3)(p_2\cdot p_3)} |\mathcal {M}^\mathrm{tree}|^2(1_g,2_g), \end{aligned}$$

where $C_A = N_c=3$ is the *SU*(3) color factor and $g_{s,b}$ is the bare strong coupling.

The factorization formula Eq. (3.5) allows us to extract contributions of soft singularities from the decay rate. To do so, we introduce the soft operator $S_3$ that extracts the most singular contributions in the soft limit from the matrix element squared and the relevant phase space:

3.6 $$\begin{aligned}&\left\langle S_3 \tilde{s}_{12}F_\mathrm{LM}(1_g,2_g,3_g)\right\rangle _\delta \nonumber \\&\quad = \frac{1}{2m_{H}}\frac{1}{3!}\int [df_{1}][df_{2}] (2\pi )^d \delta ^{(d)}\nonumber \\&\qquad \times (p_Q-p_1-p_2)|\mathcal {M}^\mathrm{tree}|^2(1_g,2_g) \nonumber \\&\qquad \times (2 C_A g_{s,b}^2)\int \frac{d^{d-1} p_3}{(2\pi )^{d-1}2E_3} \theta (E_\mathrm{max}-E_3)\nonumber \\&\qquad \times \frac{p_1\cdot p_2}{(p_1\cdot p_3)(p_2\cdot p_3)}, \end{aligned}$$

Note that the function $\theta (E_\mathrm{max} - E_3)$ prevents the integral over $E_3$ from becoming unbounded from above. We rewrite Eq. (3.6) as

3.7 $$\begin{aligned} \left\langle S_3 \tilde{s}_{12}F_\mathrm{LM}(1_g,2_g,3_g)\right\rangle _\delta =\frac{1}{3} \; \left\langle \left\langle S_3\right\rangle \; F_\mathrm{LM}(1_g,2_g)\ \right\rangle _\delta , \end{aligned}$$

where we defined^3^

3.8 $$\begin{aligned} \left\langle S_3\right\rangle\equiv & {} (2 C_A g_{s,b}^2)\int \frac{d^{d-1} p_3}{(2\pi )^{d-1}2E_3} \theta (E_\mathrm{max}-E_3)\nonumber \\&\quad \times \frac{p_1\cdot p_2}{(p_1\cdot p_3)(p_2 \cdot p_3)}\nonumber \\= & {} \frac{2 C_A [\alpha _s]}{\epsilon ^2}\left( \frac{m_{H}^2}{\mu ^2}\right) ^{-\epsilon } (\eta _{12})^{-\epsilon }\nonumber \\&\quad \times \left[ 1 + \epsilon ^2 \big [\text {Li}_2(1-\eta _{12})-\zeta _2\big ] + \mathcal {O}(\epsilon ^3) \right] , \end{aligned}$$

together with

3.9 $$\begin{aligned}{}[\alpha _s]= \frac{\alpha _s(\mu )}{2\pi }\frac{e^{\epsilon \gamma _E}}{\Gamma (1-\epsilon )}, \end{aligned}$$

and

3.10 $$\begin{aligned} \eta _{ij} = \frac{1-\cos \theta _{ij}}{2}. \end{aligned}$$

We note that in the $H \rightarrow gg$ decay discussed here $\eta _{12} = 1$; however, we do not use this fact right away and write Eq. (3.8) in a more general way. The calculation that we just described allows us to remove the soft singularity. We obtain

3.11 $$\begin{aligned}&3 \left\langle \tilde{s}_{12}F_\mathrm{LM}(1_g,2_g,3_g)\right\rangle _\delta \nonumber \\&\quad =\left\langle \left\langle S_3\right\rangle F_\mathrm{LM}(1_g,2_g)\right\rangle _\delta + 3 \left\langle (I-S_3)\tilde{s}_{12}F_\mathrm{LM}(1_g,2_g,3_g)\right\rangle _\delta . \end{aligned}$$

We note that, since the reduced matrix element does not require further regularization, all singularities in the first term on the r.h.s. of Eq. (3.11) are explicit. The second term there is free of soft singularities, but it still contains collinear ones; these occur when $\eta _{31}=(1-\cos \theta _{31})/2$ or $\eta _{32} =(1-\cos \theta _{32})/2$ vanish. To isolate these singularities, we partition the phase space in such a way that only one of them can occur at a time. To this end, we introduce the partition of unity

3.12 $$\begin{aligned} 1 = \omega ^{31} + \omega ^{32}, \end{aligned}$$

such that $\left\langle \omega ^{31}(I-S_3)\tilde{s}_{12}F_\mathrm{LM}(1_g,2_g,3_g)\right\rangle$ only has collinear singularities if $\eta _{31}\rightarrow 0$ and $\left\langle \omega ^{32}(I-S_3)\tilde{s}_{12}F_\mathrm{LM}(1_g,2_g,3_g)\right\rangle$ only has collinear singularities if $\eta _{32}\rightarrow 0$. For example, one can choose^4^

3.13 $$\begin{aligned} \omega ^{31} =\frac{\eta _{32}}{\eta _{31}+\eta _{32}},\;\;\;\; \omega ^{32} =\frac{\eta _{31}}{\eta _{31}+\eta _{32}}. \end{aligned}$$

Introducing this angular partitioning, we write

3.14 $$\begin{aligned}&\left\langle (I-S_3)\tilde{s}_{12}F_\mathrm{LM}(1_g,2_g,3_g)\right\rangle _\delta \nonumber \\&\quad =\sum \limits _{i=1}^{2}\left\langle \omega ^{3i}(I-S_3)\tilde{s}_{12}F_\mathrm{LM}(1_g,2_g,3_g)\right\rangle _\delta , \end{aligned}$$

and consider the two terms in the sum separately. We start with the $i=1$ term. Similarly to the soft case, we introduce a $C_{31}$ operator which extracts the corresponding collinear singularity, and apply it to $\left\langle \omega ^{31}(I-S_3)\tilde{s}_{12}F_\mathrm{LM}(1_g,2_g, 3_g)\right\rangle$. We define $C_{31}$ in such a way that it extracts the leading $\eta _{31 } \rightarrow 0$ singularity from $\left\langle F_\mathrm{LM}(\ldots ) \right\rangle _\delta$ without acting on the phase-space elements $[df_{1,.,3}]$, see Ref. [33] for more details. We find

3.15 $$\begin{aligned}&\left\langle C_{31}\omega ^{31}\tilde{s}_{12}F_\mathrm{LM}(1_g,2_g,3_g)\right\rangle _\delta \nonumber \\&\quad \equiv \frac{1}{2 m_{H}}\frac{1}{3!} \int [df_{1}][df_{2}][df_{3}] (2\pi )^d \delta ^{(d)}(p_Q-p_2-p_{13}) \nonumber \\&\qquad \times \left( \frac{E_1}{E_1+E_3}\right) \frac{g_{s,b}^2}{p_1\cdot p_3} P_{gg}\left( \frac{E_1}{E_{13}}\right) \otimes |\mathcal {M}^\mathrm{tree}|^2(13_g,2_g). \end{aligned}$$

In Eq. (3.15), we defined

3.16 $$\begin{aligned} p_{13} \equiv \frac{E_{13}}{E_1}p_1,~~~ E_{13} = E_{1}+E_3, \end{aligned}$$

and denoted an on-shell gluon with momentum $p_{13}$ as $13_g$. The function $P_{gg}$ in Eq. (3.15) stands for the $g^* \rightarrow gg$ splitting function and we used the $\otimes$-sign to indicate its spin-correlated product with the matrix element squared, see Refs. [30, 33] for details. In these references, we explicitly showed that at NLO spin correlations disappear after azimuthal averaging. As the result, Eq. (3.15) becomes3.17where $\left\langle P_{gg}\right\rangle$ is the spin-averaged $g^* \rightarrow gg$ splitting function

3.18 $$\begin{aligned} \left\langle P_{gg}\right\rangle (z) = 2C_A\left[ \frac{1-z}{z}+\frac{z}{1-z} + z(1-z)\right] . \end{aligned}$$

The term on the second line of Eq. (3.17) is very similar to $\left\langle F_\mathrm{LM}(1_g,2_g)\right\rangle _\delta$. To make this similarity explicit, we change integration variables from $E_1$ and $E_3$ to $E_{13}$ and $z = E_1/E_{13}$. We obtain

3.19 $$\begin{aligned} E_1= & {} z E_{13},~ E_3 = (1-z) E_{13} \Rightarrow \frac{[df_{1}] E_3^{-2\epsilon } \mathrm{d}E_3}{E_{13}}\nonumber \\= & {} [df_{13}] z \big [z(1-z)\big ]^{-2\epsilon } E_{13}^{-2\epsilon }\mathrm{d}z. \end{aligned}$$

We also rename $f_{13}$ back to $f_1$ and obtain3.20where $E_1 = m_{H}/2$ and we used the fact that the integration over *z* starts at $z=z_\mathrm{min} = \mathrm{min}\{0,1 - E_\mathrm{max}/E_{1}\}$. Since $E_\mathrm{max}$ must be chosen in such a way that the whole phase space is covered, $E_\mathrm{max}$ should be larger than $E_1$, $E_\mathrm{max} >E_1$, for all $E_1$. This implies $z_\mathrm{min}= 0$.

Repeating these steps for the soft-collinear term $S_3 C_{31}$, we find

3.21 $$\begin{aligned}&\left\langle S_3 C_{31}\omega ^{31}\tilde{s}_{12}F_\mathrm{LM}(1_g,2_g,3_g)\right\rangle _\delta \nonumber \\&\quad = \frac{1}{3} \left\langle F_\mathrm{LM}(1_g,2_g)\right\rangle _\delta \nonumber \\&\qquad \times \left[ -\frac{[\alpha _s]}{\epsilon }\frac{\Gamma ^2(1-\epsilon )}{\Gamma (1-2\epsilon )} \left( \frac{m_{H}^2}{\mu ^2}\right) ^{-\epsilon }\int \limits _{0}^1 \frac{2C_A}{(1-z)^{1+2\epsilon }}\mathrm{d}z\right] . \end{aligned}$$

We use these results to write

3.22 $$\begin{aligned}&\left\langle w^{31}(I-S_3) \tilde{s}_{12}F_\mathrm{LM}(1_g,2_g,3_g)\right\rangle _\delta \nonumber \\&\quad = \frac{1}{3} \left\langle C_{31}(I-S_3) \right\rangle \times \left\langle F_\mathrm{LM}(1_g,2_g)\right\rangle _\delta \nonumber \\&\qquad + \left\langle (I - C_{31}) w^{31}(I-S_3) \tilde{s}_{12}F_\mathrm{LM}(1_g,2_g,3_g)\right\rangle _\delta ,\nonumber \\ \end{aligned}$$

where $\left\langle C_{31}(I-S_3) \right\rangle$ follows from Eqs. (3.20, 3.21). We find

3.23 $$\begin{aligned} \left\langle C_{31}(I-S_3)\right\rangle =\frac{[\alpha _s]}{\epsilon }\frac{\Gamma ^2(1-\epsilon )}{\Gamma (1-2\epsilon )}\left( \frac{m_{H}^2}{\mu ^2}\right) ^{-\epsilon }\gamma _{z,g \rightarrow gg}^{22}\nonumber \\ \end{aligned}$$

where $\gamma _{z,g \rightarrow gg}^{22}$ is a particular case of a general anomalous dimension defined as follows

3.24 $$\begin{aligned}&\gamma ^{n k }_{f(z),g\rightarrow gg}\nonumber \\&\quad = - \int \limits _0^1 \mathrm{d}z\bigg [z^{-n \epsilon }(1-z)^{-k \epsilon } \left\langle f(z) P_{gg}(z)\right\rangle \nonumber \\&\qquad \quad - 2C_Af(1) (1-z)^{-1-k\epsilon } \bigg ]. \end{aligned}$$

We note that in the first term on the right hand side in Eq. (3.22) all singularities are manifest and the reduced matrix element does not require regularization, whereas the second term is free of *both* soft and collinear singularities so that it can be immediately integrated in four dimensions.

We deal with the $\omega ^{32}$ term in the partition of unity Eq. (3.12) in a similar way. We obtain

3.25 $$\begin{aligned}&\left\langle w^{32}(I-S_3) \tilde{s}_{12}F_\mathrm{LM}(1_g,2_g,3_g)\right\rangle _\delta \nonumber \\&\quad = \frac{1}{3} \left\langle C_{32}(I-S_3) \right\rangle \times \left\langle F_\mathrm{LM}(1_g,2_g)\right\rangle _\delta \nonumber \\&\qquad + \left\langle (I-C_{32}) w^{32}(I-S_3) \tilde{s}_{12}F_\mathrm{LM}(1_g,2_g,3_g)\right\rangle _\delta ,\nonumber \\ \end{aligned}$$

with

3.26 $$\begin{aligned}&\left\langle C_{32}(I-S_3)\right\rangle \nonumber \\&\quad = \frac{[\alpha _s]}{\epsilon }\frac{\Gamma ^2(1-\epsilon )}{\Gamma (1-2\epsilon )} \left( \frac{m_{H}^2}{\mu ^2}\right) ^{-\epsilon } \gamma _{z,g \rightarrow gg}^{22} \nonumber \\&\quad = \left\langle C_{31}(I-S_3)\right\rangle . \end{aligned}$$

We combine Eqs. (3.11, 3.23, 3.25) and obtain the following result for the three-gluon contribution to Higgs boson decay

3.27 $$\begin{aligned}&\left\langle F_\mathrm{LM}(1_g,2_g,3_g)\right\rangle _\delta \nonumber \\&\quad =\bigg [\left\langle S_3\right\rangle + 2 \left\langle C_{31}(I-S_3)\right\rangle \bigg ] \times \left\langle F_\mathrm{LM}(1_g,2_g)\right\rangle _\delta \nonumber \\&\qquad + 3 \sum _{i=1,2} \left\langle (I-C_{3i})\omega ^{3i}(I-S_3) \tilde{s}_{12}F_\mathrm{LM}(1_g,2_g,3_g)\right\rangle _\delta . \end{aligned}$$

We note that, thanks to Bose symmetry, the two terms in the sum in the last line in Eq. (3.27) are the same. Hence, we write

3.28 $$\begin{aligned}&\left\langle F_\mathrm{LM}(1_g,2_g,3_g)\right\rangle _\delta \nonumber \\&\quad =\bigg [\left\langle S_3\right\rangle + 2 \left\langle C_{31}(1-S_3)\right\rangle \bigg ] \times \left\langle F_\mathrm{LM}(1_g,2_g)\right\rangle _\delta \nonumber \\&\qquad + 6 \left\langle (I-C_{31})\omega ^{31}(I-S_3) \tilde{s}_{12}F_\mathrm{LM}(1_g,2_g,3_g)\right\rangle _\delta .\nonumber \\ \end{aligned}$$

This discussion implies that Bose symmetry can be efficiently used to partition the phase space in such a way that identical kinematic configurations of the three-gluon final states are accounted for only once in the calculation; this removes the original 1 / 3! symmetry factor.

Before combining this result with virtual corrections, we consider the other real-emission term in Eq. (3.1), $n_f\left\langle F_\mathrm{LM}(1_q,2_g,3_{\bar{q}})\right\rangle$, that describes the decay $H \rightarrow (g^* \rightarrow q \bar{q}) g$. Because (in this section) the $q \bar{q}$ pair does not directly couple to the Higgs boson, the singularity in this case is produced by the collinear splitting $g^*\rightarrow q{\bar{q}}$. For this reason, we do not need any partitioning. We repeat steps that led to Eq. (3.22) and obtain^5^

3.29 $$\begin{aligned}&n_f\left\langle F_\mathrm{LM}(1_q,2_g,3_{\bar{q}})\right\rangle _\delta \nonumber \\&\quad =n_f\left\langle (I-C_{31})F_\mathrm{LM}(1_q,2_g,3_{\bar{q}})\right\rangle _\delta \nonumber \\&\qquad +2n_f\frac{[\alpha _s]}{\epsilon } \frac{\Gamma ^2(1-\epsilon )}{\Gamma (1-2\epsilon )} \left( \frac{m_{H}^2}{\mu ^2}\right) ^{-\epsilon } \gamma _{1,g \rightarrow q \bar{q}}^{22} \;\left\langle F_\mathrm{LM}(1_g,2_g)\right\rangle _\delta , \end{aligned}$$

where

3.30 $$\begin{aligned}&\gamma _{1,g \rightarrow q \bar{q}}^{22} = -\int \limits _0^1 \mathrm{d}z \big [z(1-z)\big ]^{-2\epsilon } \left\langle P_{gq}\right\rangle (z),\nonumber \\&\quad \left\langle P_{gq}\right\rangle (z) = T_R\left[ 1 - \frac{2z (1-z)}{1-\epsilon }\right] . \end{aligned}$$

We can now combine the $H\rightarrow ggg$ and $H\rightarrow qg{\bar{q}}$ decay channels and write the total real-emission contribution to $\mathrm{d}\Gamma ^\mathrm{NLO}$, up to higher orders in $\epsilon$, as

3.31 $$\begin{aligned}&\left\langle F_\mathrm{LM}(1,2,3)\right\rangle _\delta \nonumber \\&\quad = \left\langle F_\mathrm{LM}(1_g,2_g,3_g)\right\rangle _\delta \nonumber \\&\qquad + n_f\left\langle F_\mathrm{LM}(1_q,2_g,3_{\bar{q}})\right\rangle _\delta =[\alpha _s]\left( \frac{m_{H}^2}{\mu ^2}\right) ^{-\epsilon }\nonumber \\&\qquad \times \Bigg ( \frac{2 C_A}{\epsilon ^2} \bigg [1+\epsilon ^2\big [\text {Li}_2(1-\eta _{12})-\zeta _2 \big ] \bigg ]\nonumber \\&\qquad + \frac{2 \gamma _{g}(\epsilon )}{\epsilon } +\mathcal {O}(\epsilon ) \Bigg ) \langle F_\mathrm{LM}(1_g,2_g) \rangle _\delta \nonumber \\&\qquad + 6 \left\langle (I-C_{31})\omega ^{31}(I-S_3) \tilde{s}_{12}F_\mathrm{LM}(1_g,2_g,3_g)\right\rangle _\delta \nonumber \\&\qquad + n_f\left\langle (I-C_{31})F_\mathrm{LM}(1_q,2_g,3_{\bar{q}})\right\rangle _\delta , \end{aligned}$$

where we have defined

3.32 $$\begin{aligned} \gamma _g(\epsilon ) = \gamma ^{22}_{z,g \rightarrow gg} + n_f\gamma ^{22}_{1,g \rightarrow q \bar{q}}(\epsilon ) = \gamma _g + \epsilon \gamma _g' + {\mathcal {O}}(\epsilon ^2). \end{aligned}$$

The two quantities $\gamma _g$ and $\gamma _g'$ are given in Eq. (A.7).

It remains to combine Eq. (3.31) with virtual corrections. We follow Ref. [35] to separate the divergent and finite parts of the one-loop amplitude and define

3.33 $$\begin{aligned} \left\langle F_\mathrm{LV}(1_g,2_g)\right\rangle _\delta= & {} \left\langle F_{\mathrm{LV}}^\mathrm{fin}(1_g,2_g)\right\rangle _\delta \nonumber \\&-2 [\alpha _s]\cos (\epsilon \pi ) C_A \left( \frac{1}{\epsilon ^2} + \frac{\gamma _g}{C_{A}\epsilon }\right) \nonumber \\&\times \left\langle \left( \frac{4 E_1 E_2 \eta _{12}}{\mu ^2}\right) ^{-\epsilon } F_\mathrm{LM}(1_g,2_g) \right\rangle _\delta ,\nonumber \\ \end{aligned}$$

where $\left\langle F_{\mathrm{LV}}^\mathrm{fin}(1_g,2_g)\right\rangle _\delta$ is a finite remainder of the one-loop $H \rightarrow gg$ amplitude, see Appendix A in Ref. [33] for details. We combine Eq. (3.31) and Eq. (3.33), use $\eta _{12} = 1$ and obtain a very simple result for the NLO QCD corrections to $H \rightarrow gg$ decay. It reads

3.34 $$\begin{aligned}&\mathrm{d}\Gamma ^\mathrm{NLO}_{Q \rightarrow 2} \nonumber \\&\quad = \frac{\alpha _s(\mu )}{2\pi }\left( 2 \gamma '_g +\frac{2\pi ^2}{3}C_A\right) \left\langle F_\mathrm{LM}(1_g,2_g)\right\rangle _\delta +\left\langle F_{\mathrm{LV}}^\mathrm{fin}(1_g,2_g)\right\rangle _\delta ,\nonumber \\&\mathrm{d}\Gamma ^\mathrm{NLO}_{Q\rightarrow 3} \nonumber \\&\quad = \langle (I-C_{31}) ( 6 \omega ^{31}(I-S_3) \tilde{s}_{12}F_\mathrm{LM}(1_g,2_g,3_g)\nonumber \\&\qquad + n_f F_{\mathrm{LM},{(}}1_q,2_g,3_{\bar{q}}) ) \rangle _\delta , \end{aligned}$$

where the two contributions are defined in Eq. (2.9).

We conclude this section by reminding the reader that the NLO construction we just described is identical to the FKS subtraction scheme [36, 37]. In the next sections, we will show how to generalize the FKS scheme to NNLO.

## Higgs decay to gluons: a NNLO computation

In this section we generalize the discussion of the NLO QCD corrections to the decay of a color singlet to the NNLO case. We will follow Refs. [30, 33] and perform subtractions of soft and collinear divergences in an iterated manner, starting from the soft ones. Many technical details are similar to the production case described at length in the above references and we do not discuss them here. Instead, we focus on the peculiarities of the decay.

### Double-real contribution

There are four different partonic final states that we have to consider. They are *a*) 4 gluons, *b*) 2 gluons, 2 quarks, *c*) two quark pairs of different flavors and *d*) two quark pairs of the same flavor. We write

4.1 $$\begin{aligned}&\left\langle F_\mathrm{LM}(1,2,3,4)\right\rangle _\delta \nonumber \\&\quad = \left\langle F_\mathrm{LM}(1_g,2_g,3_g,4_g)\right\rangle _\delta +n_f\left\langle F_\mathrm{LM}(1_g,2_g,3_q,4_{\bar{q}})\right\rangle _\delta \nonumber \\&\qquad + \frac{n_f(n_f-1)}{2}\left\langle F_\mathrm{LM}(1_q,2_{q'},3_{\bar{q}},4_{{\bar{q}}'})\right\rangle _\delta \nonumber \\&\qquad +n_f\left\langle F_\mathrm{LM}(1_q,2_q,3_{\bar{q}},4_{\bar{q}})\right\rangle _\delta . \end{aligned}$$

In full analogy to the NLO case, we partition the phase space in such a way that only a subset of partons are allowed to become unresolved. In case of the NNLO contributions, *two* partons can become unresolved simultaneously; we will systematically rename partons so that, eventually, the unresolved partons are always referred to as $f_3$ and $f_4$.

We first consider the four-gluon channel,

4.2 $$\begin{aligned} H \rightarrow g(p_1) g(p_2) g(p_3) g(p_4), \end{aligned}$$

and introduce a partition of unity following what has already been done at NLO

4.3 $$\begin{aligned} 1=\tilde{s}_{12}+\tilde{s}_{13}+\tilde{s}_{14}+\tilde{s}_{23} +\tilde{s}_{24}+\tilde{s}_{34}. \end{aligned}$$

We insert this partition inside the integrand for $\left\langle F_\mathrm{LM}(1_g,2_g,3_g,4_g)\right\rangle _\delta$, use the symmetry of the phase space and the matrix element and arrive at^6^

4.4 $$\begin{aligned}&2m_{H}\left\langle F_\mathrm{LM}(1_g,2_g,3_g,4_g)\right\rangle \nonumber \\&\quad =\frac{1}{4!}\int \prod \limits _{i=1}^{4}[df_i]\;(2\pi )^d\delta ^{(d)}\nonumber \\&\qquad \times \left( p_Q - \sum \limits _{i=1}^{4} p_i\right) \sum \limits _{i \ne j=1}^{4} \tilde{s}_{ij} |{\mathcal {M}}(1_g,2_g,3_g,4_g)|^2\nonumber \\&\quad =\frac{1}{4}\int \prod \limits _{i=1}^{4}[df_i]\;(2\pi )^d \delta ^{(d)}\nonumber \\&\qquad \times \left( p_Q - \sum \limits _{i=1}^{4} p_i\right) \; \tilde{s}_{12} \; |{\mathcal {M}}(1_g,2_g,3_g,4_g)|^2. \end{aligned}$$

The prefactor ${\tilde{s}}_{12}$ ensures that no singularity arises in the product $\tilde{s}_{12} \; |M(1_g,2_g,3_g,4_g)|^2$ when gluons 1 and 2 become either soft or collinear to each other. To proceed further, we introduce an energy ordering for potentially-unresolved gluons $g_3$ and $g_4$, use $g_3 \leftrightarrow g_4$ symmetry and write

4.5 $$\begin{aligned}&2m_{H}\left\langle F_\mathrm{LM}(1_g,2_g,3_g,4_g)\right\rangle \nonumber \\&\quad =\frac{1}{2}\int \prod \limits _{i=1}^{4}[df_i]\;(2\pi )^d\delta ^{(d)}\nonumber \\&\qquad \times \left( p_Q - \sum \limits _{i=1}^{4} p_i\right) \tilde{s}_{12} \theta (E_3-E_4) \nonumber \\&\qquad \times |{\mathcal {M}}(1_g,2_g,3_g,4_g)|^2 \nonumber \\&\quad = 12{\times } (2 m_H) \langle \left\langle \tilde{s}_{12}F_\mathrm{LM}(1_g,2_g,3_g,4_g) \theta (E_3 {-} E_4)\right\rangle . \end{aligned}$$

We now consider the 2*q*2*g* final state. In principle, it contains fewer singularities than the four-gluon final state. Therefore, one may use a simpler partition of unity to single out the potentially unresolved partons. However, to streamline the bookkeeping, we find it convenient to use identical partitioning for all final states. Our starting point is then

4.6 $$\begin{aligned}&2m_{H}\left\langle F_\mathrm{LM}(1_g,2_g,3_q,4_{\bar{q}}) \right\rangle \nonumber \\&\quad =\frac{1}{2!}\int \prod \limits _{i=1}^{4}[df_i]\;(2\pi )^d\delta ^{(d)}\nonumber \\&\qquad \times \left( p_Q - \sum \limits _{i=1}^{4} p_i\right) \sum \limits _{i \ne j =1}^{4} \tilde{s}_{ij} |{\mathcal {M}}(1_g,2_g,3_q,4_{\bar{q}})|^2,\nonumber \\ \end{aligned}$$

where the partition of unity Eq. (4.3) has already been employed. We note that the amplitude is symmetric with respect to permutations of the two gluons, so that

4.7 $$\begin{aligned} |{\mathcal {M}}(i_g,j_g, k_q,l_{\bar{q}})|^2 = |{\mathcal {M}}(j_g,i_g, k_q,l_{\bar{q}})|^2. \end{aligned}$$

Furthermore, since in this amplitude the quark-antiquark pair arises from gluon splitting, the amplitude squared summed over quark and anti-quark polarizations satisfies $|{\mathcal {M}}(i_g,j_g,k_q, l_{\bar{q}})|^2=|{\mathcal {M}}(i_g,j_g,l_q,k_{\bar{q}})|^2$. We can use these symmetries of the amplitude squared as well as the symmetry of the phase space to re-write Eq. (4.6) in the following way

4.8 $$\begin{aligned}&2m_{H}\left\langle F_\mathrm{LM}(1_g,2_g,3_q,4_{\bar{q}}) \right\rangle \nonumber \\&\quad =\frac{1}{2!}\int \prod \limits _{i=1}^{4}[df_i]\;(2\pi )^d \delta ^{(d)}\nonumber \\&\qquad \times \left( p_Q - \sum \limits _{i=1}^{4} p_i\right) \nonumber \\&\qquad \times \tilde{s}_{12} \biggl ( |{\mathcal {M}}(1_g,2_g,3_q,4_{\bar{q}})|^2 +|{\mathcal {M}}(1_g,3_g,2_q,4_{\bar{q}})|^2\nonumber \\&\qquad +|{\mathcal {M}}(1_g,4_g,2_q,3_{\bar{q}})|^2+|{\mathcal {M}}(2_g,3_g,1_q,4_{\bar{q}})|^2 \nonumber \\&\qquad +|{\mathcal {M}}(2_g,4_g,1_q,3_{\bar{q}})|^2+|{\mathcal {M}}(3_g,4_g,1_q,2_{\bar{q}})|^2 \biggr ).\nonumber \\ \end{aligned}$$

To proceed further, we introduce the energy ordering for the two potentially unresolved partons $f_{3,4}$ and use symmetries of the amplitude to remove the factor 1 / 2 in the above equation. In cases when $f_{3}$ and $f_{4}$ are partons of a different type, this requires us to combine the different contributions in a particular way. As an example, consider the second and the third term in Eq. (4.8). Relabelling parton momenta where appropriate, we write

4.9 $$\begin{aligned}&|{\mathcal {M}}(1_g,3_g,2_q,4_{\bar{q}})|^2 +|{\mathcal {M}}(1_g,4_g,2_q,3_{\bar{q}})|^2\nonumber \\&\quad = \big [ |{\mathcal {M}}(1_g,3_g,2_q,4_{\bar{q}})|^2 +|{\mathcal {M}}(1_g,4_g,2_q,3_{\bar{q}})|^2 \nonumber \\&\qquad + |{\mathcal {M}}(1_g,4_g,2_q,3_{\bar{q}})|^2\nonumber \\&\qquad + |{\mathcal {M}}(1_g,3_g,2_q,4_{\bar{q}})|^2 \big ] \theta (E_3 - E_4) \nonumber \\&\quad = 2 \left[ |{\mathcal {M}}(1_g,3_g,2_q,4_{\bar{q}})|^2 \right. \nonumber \\&\qquad \left. + |{\mathcal {M}}(1_g,4_g,2_q,3_{\bar{q}})|^2 \right] \theta (E_3 - E_4). \end{aligned}$$

Using these transformations, we obtain

4.10 $$\begin{aligned}&2m_{H}\left\langle F_\mathrm{LM}(1_g,2_g,3_q,4_{\bar{q}}) \right\rangle \nonumber \\&\quad = \int \prod \limits _{i=1}^{4} [df_i] \;(2\pi )^d \delta ^{(d)} \left( p_Q - \sum \limits _{i=1}^{4} p_i\right) \; \theta (E_3 - E_4)\nonumber \\&\qquad \times \tilde{s}_{12} \bigl ( |{\mathcal {M}}(1_g,2_g, 3_q,4_{\bar{q}})|^2 +|{\mathcal {M}}(1_g,3_g,2_q,4_{\bar{q}})|^2\nonumber \\&\qquad +|{\mathcal {M}}(1_g,4_g,2_q,3_{\bar{q}})|^2 +|{\mathcal {M}}(2_g,3_g,1_q,4_{\bar{q}})|^2\nonumber \\&\qquad +|{\mathcal {M}}(2_g,4_g,1_q,3_{\bar{q}})|^2 +|{\mathcal {M}}(3_g,4_g,1_q,2_{\bar{q}})|^2 \bigr ),\nonumber \\ \end{aligned}$$

which we can write as

4.11 $$\begin{aligned}&\left\langle F_\mathrm{LM}(1_g,2_g,3_q,4_{\bar{q}}) \right\rangle \nonumber \\&\quad =2 \bigg \langle \bigg [ F_\mathrm{LM}(1_g,2_g,3_q,4_{\bar{q}}) + F_\mathrm{LM}(1_g,3_g,2_q,4_{\bar{q}})\nonumber \\&\qquad + F_\mathrm{LM}(1_g,4_g,2_q,3_{\bar{q}}) + F_\mathrm{LM}(2_g,3_g,1_q,4_{\bar{q}})\nonumber \\&\qquad + F_\mathrm{LM}(2_g,4_g,1_q,3_{\bar{q}}) + F_\mathrm{LM}(3_g,4_g,1_q,2_{\bar{q}})\bigg ] \tilde{s}_{12}\theta (E_3-E_4) \bigg \rangle . \end{aligned}$$

We note that the six terms in Eq. (4.11) have very different singularity structures. For example, all the terms in Eq. (4.11) that contain gluon $g_4$ give rise to single soft singularities that arise when $E_4 \rightarrow 0$. In the remaining three terms, the energy $E_4$ is associated with an anti-quark and, therefore, these terms are not singular in the single-soft limit. Similarly, the collinear limit $C_{41}$ corresponds to an (anti)quark and a gluon becoming collinear in the first, second, fifth and sixth terms in Eq. (4.11). However, the same limit describes a kinematic configuration with two collinear gluons in the third term in Eq. (4.11). Clearly, the two limiting cases result in different splitting functions and different reduced matrix elements.

Finally, we turn to the four-quark channels, where we need to make a further distinction between cases when quarks have same or different flavors. If they are different, i.e. $q \ne q'$, we write

4.12 $$\begin{aligned}&2m_{H}\left[ \frac{n_f(n_f-1)}{2}\right] \left\langle F_\mathrm{LM}(1_q,2_{q'},3_{\bar{q}},4_{{\bar{q}}'} ) \right\rangle \nonumber \\&\quad = \frac{n_f(n_f-1)}{2} \int \prod \limits _{i=1}^{4}[df_i] \;(2\pi )^d \delta ^{(d)}\nonumber \\&\qquad \times (p_Q - \sum \limits _{i=1}^{4} p_i) \; |{\mathcal {M}}(1_q,2_{q'},3_{{\bar{q}}},4_{{\bar{q}}'})|^2. \end{aligned}$$

If the flavors are identical, we can use the same amplitude ${\mathcal {M}}$ as for the different-flavor case, accounting for a permutation of two identical particles. We write

4.13 $$\begin{aligned}&2m_{H}\;n_f\; \langle F_\mathrm{LM}(1_q,2_{q},3_{\bar{q}},4_{{\bar{q}}}) \rangle \nonumber \\&\quad = \frac{n_f}{(2!)^2} \int \prod \limits _{i=1}^{4} [df_i] \;(2\pi )^d \delta ^{(d)}\left( p_Q - \sum \limits _{i=1}^{4} p_i\right) \nonumber \\&\qquad \times |{\mathcal {M}}(1_q,2_{q'},3_{\bar{q}},4_{{\bar{q}}'}) - {\mathcal {M}} (1_q,2_{q'},4_{\bar{q}},3_{{\bar{q}}'})|^2.\nonumber \\ \end{aligned}$$

We denote the interference term as

4.14 $$\begin{aligned}&\mathrm{Int}(1_q,2_q,3_{\bar{q}},4_{\bar{q}}) \nonumber \\&\quad = -2\mathrm{Re}\left( {\mathcal {M}}(1_q,2_{q'},3_{\bar{q}},4_{{\bar{q}}'}) {\mathcal {M}}^*(1_q,2_{q'},4_{\bar{q}},3_{{\bar{q}}'}) \right) ,\nonumber \\ \end{aligned}$$

and write the *complete* four-quark contribution to the decay rate, including both different and identical flavors, as

4.15 $$\begin{aligned}&2m_{H}\langle F_\mathrm{LM}^{(4q)}(1,2,3,4) \rangle \nonumber \\&\quad = \frac{n_f^2}{2} \int \prod \limits _{i=1}^{4}[df_i]\;(2\pi )^d \delta ^{(d)}\nonumber \\&\qquad \times \left( p_Q - \sum \limits _{i=1}^{4} p_i\right) |{\mathcal {M}}(1_q,2_{q'},3_{{\bar{q}}},4_{{\bar{q}}'})|^2 \nonumber \\&\qquad + \frac{n_f}{4} \int \prod \limits _{i=1}^{4}[df_i]\;(2\pi )^d \delta ^{(d)}\nonumber \\&\qquad \times \left( p_Q - \sum \limits _{i=1}^{4} p_i\right) \mathrm{Int}(1,2,3,4). \end{aligned}$$

The interference term in Eq. (4.15) is not singular and can be evaluated in four dimensions; for this reason we keep it as it is. Moreover, the first term in that equation only produces singularities when either one or two $q{\bar{q}}$ pairs become collinear. Despite this simplicity, we find it convenient to treat the four-quark contributions Eq. (4.15) in the same way as the two other channels that we discussed previously. To this end, we insert the partition of unity Eq. (4.3) into the integrands in Eq. (4.15), re-label partonic momenta, use the symmetry of the amplitude squared

4.16 $$\begin{aligned} |{\mathcal {M}}(i_q,j_{q'},k_{\bar{q}},l_{{\bar{q}}'})|^2= & {} |{\mathcal {M}}(i_q,l_{q'},k_{\bar{q}},j_{{\bar{q}}'})|^2 \nonumber \\= & {} |{\mathcal {M}}(k_q,j_{q'},i_{\bar{q}},l_{{\bar{q}}'})|^2, \end{aligned}$$

and obtain

4.17 $$\begin{aligned}&2m_{H}\langle F_\mathrm{LM}^{(4q)}(1,2,3,4) \rangle \nonumber \\&\quad =n_f^2 \int \prod \limits _{i=1}^{4} [d f_i] \;(2\pi )^d \delta ^{(d)}\left( p_Q - \sum \limits _{i=1}^{4} p_i\right) \nonumber \\&\qquad \times \tilde{s}_{12}\biggl ( 2 |{\mathcal {M}}(1_q,2_{q'},3_{\bar{q}},4_{{\bar{q}}'} )|^2 + |{\mathcal {M}}(1_q,3_{q'},2_{{\bar{q}}},4_{{\bar{q}}'})|^2 \biggr ) \nonumber \\&\qquad + \frac{n_f}{4} \int \prod \limits _{i=1}^{4} [d f_i] \;(2\pi )^d \delta ^{(d)} \left( p_Q - \sum \limits _{i=1}^{4} p_i\right) \; \mathrm{Int}(1,2,3,4). \end{aligned}$$

The prospective unresolved partons are $f_{3,4}$. Similar to other channels, we introduce the energy ordering $E_3 > E_4$ and again use the symmetry of the amplitude squared to simplify the result. We obtain

4.18 $$\begin{aligned}&\langle F_\mathrm{LM}^{(4q)}(1,2,3,4) \rangle \nonumber \\&\quad = n_f\left\langle F_\mathrm{LM}^\mathrm{int} (1_q,2_{q},3_{\bar{q}},4_{{\bar{q}}}) \right\rangle \nonumber \\&\qquad + 2n_f^2 \big \langle \big [ F_\mathrm{LM}(1_q,2_{q'},3_{\bar{q}},4_{{\bar{q}}'}) + F_\mathrm{LM}(1_q,2_{q'},4_{\bar{q}},3_{{\bar{q}}'})\nonumber \\&\qquad + F_\mathrm{LM}(1_q,3_{q'},2_{\bar{q}},4_{{\bar{q}}'}) \big ]\tilde{s}_{12}\theta (E_3 - E_4) \big \rangle , \end{aligned}$$

where we have defined

4.19 $$\begin{aligned}&n_f\left\langle F_\mathrm{LM}^\mathrm{int}(1_q,2_{q},3_{\bar{q}},4_{{\bar{q}}}) \right\rangle \nonumber \\&\quad =\frac{n_f}{4} \left[ \frac{1}{2m_{H}}\right] \int \prod \limits _{i=1}^{4} [d f_i] \;(2\pi )^d \delta ^{(d)}\nonumber \\&\qquad \times \left( p_Q - \sum \limits _{i=1}^{4} p_i\right) \; \mathrm{Int}(1,2,3,4). \end{aligned}$$

Upon combining all the channels, we obtain the final result for the double-real contribution to the decay width. It reads

4.20 $$\begin{aligned}&\left\langle F_\mathrm{LM}(1,2,3,4) \right\rangle _\delta \nonumber \\&\quad =\Bigg \langle \tilde{s}_{12}\theta (E_3 - E_4)\times \Bigg \{ 12 F_\mathrm{LM}(1_g,2_g,3_g,4_g) \nonumber \\&\qquad + 2n_f\bigg [F_\mathrm{LM}(1_g,2_g,3_q,4_{\bar{q}})+F_\mathrm{LM}(1_g,3_g,2_q,4_{\bar{q}})\nonumber \\&\qquad +F_\mathrm{LM}(1_g,4_g,2_q,3_{\bar{q}})+F_\mathrm{LM}(2_g,3_g,1_q,4_{\bar{q}})\nonumber \\&\qquad +F_\mathrm{LM}(2_g,4_g,1_q,3_{\bar{q}})+F_\mathrm{LM}(3_g,4_g,1_q,2_{\bar{q}})\bigg ]\nonumber \\&\qquad +2n_f^2 \bigg [F_\mathrm{LM}(1_q,2_{q'},3_{\bar{q}},4_{{\bar{q}}'})+F_\mathrm{LM}(1_q,2_{q'},4_{{\bar{q}}},3_{{\bar{q}}'})\nonumber \\&\qquad +F_\mathrm{LM}(1_q,3_{q'},2_{\bar{q}},4_{{\bar{q}}'})\bigg ]\Bigg \} +n_fF_\mathrm{LM}^\mathrm{int}(1_q,2_q,3_{\bar{q}},4_{\bar{q}})\Bigg \rangle _\delta . \end{aligned}$$

To illustrate how soft and collinear singularities are extracted from the double-real emission contribution Eq. (4.20), we focus on the four-gluon final state $F_\mathrm{LM}(1_g,2_g,3_g,4_g)$. This contribution possesses the richest singularity structure yet, at the same time, it is one of the simplest as far as the bookkeeping is concerned. After explaining how the singularities are extracted in this case, we present the results for all channels in Sect. 4.4.

#### Double-soft contribution for $H \rightarrow gggg$

Similar to the production case, we begin with the double-soft limit that occurs when $E_3,E_4\rightarrow 0$. We follow Refs. [30, 33] and introduce an operator  that extracts the leading double-soft singularity from the product of the matrix element squared and the phase space, and write4.21The double-soft limit is computed in exactly the same way as in the production case [30, 33]. We find4.22where [31]4.23We use Eq. (4.21) and write4.24The term on the second line in Eq. (4.24) does not contain double-soft singularities anymore but it still contains both single-soft and collinear ones. We discuss how to extract them in what follows.

#### Single-soft contribution

We need to extract the single-soft singularity from4.25see Eq. (4.24). The soft limit of the amplitude squared reads

4.26 $$\begin{aligned}&S_4 |\mathcal {M}^\mathrm{tree}(1_g,2_g,3_g,4_g)|^2 \nonumber \\&\quad = g_{s,b}^2 C_A \sum \limits _{(ij) \in {1,2,3}}^{} \frac{p_i \cdot p_j}{(p_i \cdot p_4) ( p_j \cdot p_4)}\nonumber \\&\qquad \times |\mathcal {M}^\mathrm{tree}(1_g,2_g,3_g)|^2, \end{aligned}$$

where the sum runs over three *ij*-pairs, $\{1,2\},\{1,3\},\{2,3\}$. The gluon $g_4$ decouples both from the hard matrix element and the phase-space; hence, integration over its four-momentum is identical to the NLO case except that the upper boundary for the $E_4$ integration is now $E_3$. Repeating steps analogous to what we discussed at NLO, we find4.27where

4.28 $$\begin{aligned} J_{S_4}^{ggg}= & {} [\alpha _s]\left( \frac{4 E_3^2}{\mu ^2}\right) ^{-\epsilon } \; \frac{C_A}{\epsilon ^2}\nonumber \\&\times \Bigg [\left( \eta _{12}\right) ^{-\epsilon } K_{12} +\left( \eta _{13}\right) ^{-\epsilon } K_{13} +\left( \eta _{23}\right) ^{-\epsilon } K_{23}\Bigg ],\nonumber \\ \end{aligned}$$

and

4.29 $$\begin{aligned} K_{ij}= & {} \frac{\Gamma ^2(1-\epsilon )}{\Gamma (1-2\epsilon )} \eta _{ij}^{1+\epsilon }~_2 F_1(1,1,1-\epsilon ;1-\eta _{ij}) \nonumber \\= & {} 1 + \epsilon ^2\big [\text {Li}_2(1-\eta _{ij}) - \zeta _2\big ] + \mathcal {O}(\epsilon ^3). \end{aligned}$$

Equation (4.27) is free from soft singularities, but it still contains collinear ones; these arise when the gluon $g_3$ becomes collinear to gluon $g_1$ or gluon $g_2$. We proceed as in the NLO computation. Namely, we introduce a partition of unity, use the symmetry of the process under the exchange of gluons 1 and 2 and write

4.30 $$\begin{aligned}&\left\langle (I-S_3) J^{ggg}_{S_4} \tilde{s}_{12}F_\mathrm{LM}(1_g,2_g,3_g) \right\rangle _\delta \nonumber \\&\quad = 2 \left\langle C_{31} \omega ^{31} (I-S_3) J^{ggg}_{S_4} \tilde{s}_{12}F_\mathrm{LM}(1_g,2_g,3_g) \right\rangle _\delta \nonumber \\&\qquad +\left\langle \sum _{i=1,2} (I-C_{3i}) \omega ^{3i} (I-S_3) J^{ggg}_{S_4} \tilde{s}_{12}F_\mathrm{LM}(1_g,2_g,3_g) \right\rangle _\delta . \end{aligned}$$

All singularities are regulated in the second term on the r.h.s. of Eq. (4.30). We now consider the first term on the r.h.s. of Eq. (4.30). Taking the $\eta _{31}\rightarrow 0$ limit in $J^{ggg}_{S_4}$, we obtain

4.31 $$\begin{aligned} C_{31} J_{S_4}^{ggg}&= [\alpha _s]\left( \frac{4 E_3^2}{\mu ^2}\right) ^{-\epsilon } \frac{C_A}{\epsilon ^2}\nonumber \\&\quad \times \bigg [ 2\left( \eta _{12}\right) ^{-\epsilon } K_{12} +\frac{\Gamma ^3(1-\epsilon )\Gamma (1+\epsilon )}{\Gamma (1-2\epsilon )} \left( \eta _{31}\right) ^{-\epsilon }\bigg ], \end{aligned}$$

where we used

4.32 $$\begin{aligned} \lim _{\eta _{ij}\rightarrow 0} K_{ij} = \frac{\Gamma ^3(1-\epsilon ) \Gamma (1+\epsilon )}{\Gamma (1-2\epsilon )}. \end{aligned}$$

Since we have to apply the $C_{31}$ operator to $3 \left\langle J_{S_4}^{ggg} (I-S_3)\tilde{s}_{12}F_\mathrm{LM}(1_g,2_g,3_g)\right\rangle _\delta$ and since the limit of $F_\mathrm{LM}(1_g,2_g,3_g)$ is identical to what we already discussed in the NLO case, the computation proceeds similarly to the NLO case. Note that since the $(E_3^2)^{-\epsilon }$ prefactor in $J_{S_4}$ gives an extra factor $(1-z)^{-2\epsilon }$ the relevant anomalous dimension in this case is $\gamma ^{24}_{z,g\rightarrow gg}$, c.f. Eq. (3.24). The result of the calculation reads

4.33 $$\begin{aligned}&3\left\langle (I-S_3) J^{ggg}_{S_4} \tilde{s}_{12}F_\mathrm{LM}(1_g,2_g,3_g) \right\rangle _\delta \nonumber \\&\quad = +2\frac{[\alpha _s]^2}{\epsilon ^3} \left( \frac{\mu ^2}{m_{H}^2}\right) ^{2\epsilon }C_A\nonumber \\&\qquad \times \bigg [\frac{2\Gamma ^4(1-\epsilon )}{\Gamma ^2(1-2\epsilon )} +\frac{\Gamma ^4(1-\epsilon ) \Gamma (1+\epsilon )}{2\Gamma (1-3\epsilon )}\bigg ]\nonumber \\&\qquad \times \gamma _{z,g\rightarrow gg}^{24}\left\langle F_\mathrm{LM}(1_g,2_g)\right\rangle _\delta \nonumber \\&\qquad +3\left\langle \sum _{i=1,2} (I-C_{3i}) \omega ^{3i} (I-S_3) J^{ggg}_{S_4} \tilde{s}_{12}F_\mathrm{LM}(1_g,2_g,3_g) \right\rangle _\delta . \end{aligned}$$

We combine the different contributions and obtain4.34In Eq. (4.34) the third and fourth lines are free from unregulated singularities whereas the second line contains unregulated collinear singularities that need to be extracted. We explain how to do that in the next section.

#### Collinear singularities: general structure

Having regulated all the soft singularities, we are left with only one contribution on the right hand side of Eq. (4.34),4.35that still contains unregulated collinear singularities. To isolate and extract them, we need to introduce a partition of unity

4.36 $$\begin{aligned} 1 = w^{31,41} + w^{32,42} + w^{31,42} + w^{32,41}, \end{aligned}$$

where $w^{3i,4j}$ are functions of the partons’ emission angles. These functions are constructed in such a way that a product of $w^{3i,4j}$ with the matrix element squared has non-integrable collinear singularities if gluon $g_3$ is collinear to gluon $g_i$ or gluon $g_4$ is collinear to gluon $g_j$. The singularities that arise when gluons $g_3$ and $g_4$ become collinear can only occur in the partitions $w^{31,41}$ and $w^{32,42}$. Following Refs. [30, 33], we refer to $w^{31,41}$ and $w^{32,42}$ as the triple-collinear partitions and $w^{31,42}$ and $w^{41,32}$ as the double-collinear partitions. A possible choice for these functions is given in Appendix A.

The double-collinear partitions can be dealt with in a relatively straightforward way since the collinear singularities are clearly isolated. The only issue that we need to address is the construction of a proper phase space for this contribution; we discuss how this can be done in Appendix B. For the triple-collinear partitions, we need to order the emission angles of gluons $g_3$ and $g_4$ and we refer to these orderings as “sectors” that we label as *a, b, c, d*, see Refs. [30, 33] for details. Explicitly, we write

4.37 $$\begin{aligned} 1= & {} \theta \left( \eta _{41}< \frac{\eta _{31}}{2} \right) + \theta \left( \frac{\eta _{31}}{2}< \eta _{41}< \eta _{31} \right) \nonumber \\&+ \theta \left( \eta _{31}< \frac{\eta _{41}}{2} \right) + \theta \left( \frac{\eta _{41}}{2}< \eta _{31} < \eta _{41} \right) \nonumber \\= & {} \theta ^{(a)} + \theta ^{(b)} + \theta ^{(c)} + \theta ^{(d)}. \end{aligned}$$

Once partitions and sectors are introduced, we can extract the collinear limits from the decay rates following the procedure already discussed for the production case [30, 33]. Note, however, that similar to the NLO computations discussed in Sect. 3, we have to integrate the various splitting functions appearing in the calculation over energies to obtain (generalized) anomalous dimensions.

We now summarize the relevant steps for the extraction of the collinear singularities, closely following the procedure and notation of Ref. [30, 33]. We introduce the short-hand notation4.38and write

4.39 $$\begin{aligned} \left\langle \mathcal {G}(1,2,3,4)\right\rangle _{\delta }= & {} \left\langle \mathcal {G}^{s_r, c_s}(1,2,3,4)\right\rangle _\delta \nonumber \\&+ \left\langle \mathcal {G}^{s_r, c_t}(1,2,3,4)\right\rangle _\delta + \left\langle \mathcal {G}^{s_r, c_r}(1,2,3,4)\right\rangle _\delta .\nonumber \\ \end{aligned}$$

In Eq. (4.39), we have introduced

- the soft-regulated, single-collinear contribution
  4.40 $$\begin{aligned}&\left\langle \mathcal {G}^{s_r, c_s}(1,2,3,4)\right\rangle _\delta \nonumber \\&\quad = \sum _{(ij)\in \{12,21\}} \left\langle \bigg [C_{3i}[df_{3}] + C_{4j}[df_{4}] \bigg ]\omega ^{3i,4j} \mathcal {G}(1,2,3,4)\right\rangle _\delta \nonumber \\&\qquad + \sum _{i\in \{1,2\}} \left\langle \bigg [\theta ^{(a)} C_{4i} + \theta ^{(b)} C_{43} + \theta ^{(c)} C_{3i} + \theta ^{(d)} C_{43}\bigg ]\right. \nonumber \\&\qquad \times \left. [df_{3}][df_{4}] \omega ^{3i,4i} \mathcal {G}(1,2,3,4)\right\rangle _\delta ; \end{aligned}$$
- the soft-regulated triple- and double-collinear contribution, defined as 4.41
- and, finally, the soft-regulated collinear-regulated term 4.42

We remind the reader that the notations in Eqs. (4.40, 4.41, 4.42) are such that collinear operators act on everything that appears to the right of them. In particular, the notation $\left\langle \cdots C [df_{i}] \cdots \right\rangle$ implies that a particular collinear limit should be taken in the phase-space element of the parton $f_i$. More details can be found in Refs. [30, 33] where we show an explicit parametrization of the emission angles for gluons $g_3$ and $g_4$ and define the action of collinear operators in terms of this parametrization.

We discuss the terms $$\left\langle \mathcal {G}^{s_r,c_s}(1,2,3,4) \right\rangle $$ and $$\left\langle \mathcal {G}^{s_r,c_t}(1,2,\right. $$$$\left. 3,4)\right\rangle $$ in the next two subsections. The term $$\left\langle \mathcal {G}^{s_r, c_r}(1,2,3,4)\right\rangle $$ is finite and can be immediately computed in four dimensions. This point is again discussed in Refs. [30, 33] in the context of color singlet production. Since there is no conceptual difference between how this contribution is computed in the production and decay cases, we won’t repeat the discussion here.

#### Soft-regulated single-collinear contribution

To obtain an expression for the soft-regulated single-collinear contribution $\left\langle \mathcal {G}^{s_r,c_s}(1,2,3,4)\right\rangle$ in Eq. (4.40), we follow the same steps as in the production case [30, 33]. After a tedious but otherwise straightforward computation we obtain^7^

4.43 $$\begin{aligned}&\left\langle \mathcal {G}^{s_r,c_s}(1,2,3,4)\right\rangle _\delta \nonumber \\&\quad = \frac{[\alpha _s]}{\epsilon } \left\langle 6 \left[ \left( \frac{4 E_1^2}{\mu ^2}\right) ^{-\epsilon }\gamma _{z,g\rightarrow gg}^{22}\right. \right. \nonumber \\&\qquad \left. \left. - 2C_A\frac{\left( 4 E_4^2/\mu ^2\right) ^{-\epsilon } -\left( 4 E_1^2/\mu ^2\right) ^{-\epsilon }}{2\epsilon }\right] \right. \nonumber \\&\qquad \left. \times (I-S_3)\bigg [(I-C_{32})\tilde{\omega }_{4||1}^{32,41}\right. \nonumber \\&\qquad \left. +\left( \frac{\eta _{41}}{2}\right) ^{-\epsilon } (I-C_{31}) \tilde{\omega }^{31,41}_{4||1}\bigg ] \tilde{s}_{12}F_\mathrm{LM}(1_g,2_g,3_g)\right\rangle _\delta \nonumber \\&\qquad +\frac{[\alpha _s]}{\epsilon }\left\langle 6 \left( \frac{E_4^2}{\mu ^2}\right) ^{-\epsilon }(I-S_3)\right. \nonumber \\&\qquad \left. \times (I-C_{31}) \big [\eta _{41}^{-\epsilon }\left( 1-\eta _{41} \right) ^{\epsilon }\big ]\tilde{\omega }_{3||4}^{31,41} \tilde{s}_{12}\right. \nonumber \\&\qquad \left. \times \Bigg [\tilde{\gamma }_g(\epsilon ) F_\mathrm{LM}(1_g,2_g,3_g)\right. \nonumber \\&\qquad \left. +\epsilon \tilde{\gamma }_g(\epsilon ,k_\perp ) r_\mu r_\nu F_\mathrm{LM}^{\mu \nu } (1_g,2_g,3_g)\bigg ]\right\rangle _\delta \nonumber \\&\qquad +\frac{[\alpha _s]^2}{\epsilon ^2}\left( \frac{\mu ^2}{m_{H}^2}\right) ^{2\epsilon } \left\langle F_\mathrm{LM}(1_g,2_g)\right\rangle _\delta \left\{ 2\frac{\Gamma ^2(1-\epsilon )}{\Gamma (1-2\epsilon )}\right. \nonumber \\&\qquad \left. \times \left[ \left( \gamma _{z,g\rightarrow gg}^{22}\right) ^2 - 2C_A\left( \frac{\gamma _{z,g\rightarrow gg}^{24} -\gamma _{z,g\rightarrow gg}^{22}}{2\epsilon }\right) \right] \right. \nonumber \\&\qquad \left. +2^{\epsilon }\frac{\Gamma (1-2\epsilon )\Gamma (1-\epsilon )}{\Gamma (1-3\epsilon )}\right. \nonumber \\&\qquad \left. \times \bigg [\gamma _{z,g\rightarrow gg}^{22} \gamma _{z,g\rightarrow gg}^{42} - 2C_A\left( \frac{\gamma _{z,g\rightarrow gg}^{24}-\gamma _{z,g \rightarrow gg}^{42}}{2\epsilon }\right) \right. \nonumber \\&\left. \qquad +2^{\epsilon }\left[ \gamma _{z,g\rightarrow gg}^{24} \tilde{\gamma }_{g}(\epsilon ) + \epsilon \gamma _{z,g\rightarrow gg}^{24,r} \tilde{\gamma }_{g,k_\perp }(\epsilon )\right] \bigg ]\right. \nonumber \\&\qquad \left. -2C_A4^{\epsilon } \left[ \frac{\Gamma (1-2\epsilon )\Gamma (1-\epsilon )}{\Gamma (1-3\epsilon )} - \epsilon ^2 \Theta _{bd}\right] \delta _g(\epsilon )\right. \nonumber \\&\qquad \left. -4C_A\left[ \frac{\Gamma ^2(1-\epsilon )}{\Gamma (1-2\epsilon )} +\frac{\Gamma (1-2\epsilon )\Gamma (1-\epsilon )}{2^{1-\epsilon }\Gamma (1-3\epsilon )} -\epsilon ^2 \Theta _{ac}\right] \right. \nonumber \\&\qquad \left. \times \left( \frac{\gamma _{z,g\rightarrow gg}^{24} -\gamma _{z,g\rightarrow gg}^{22}}{2\epsilon }\right) \right\} . \end{aligned}$$

The anomalous dimensions $\gamma _{z,g\rightarrow gg}^{ij}$, that appear in Eq. (4.43), are defined in Eq. (3.24) whereas $\tilde{\gamma }_g(\epsilon )$, $\tilde{\gamma }_{g}(\epsilon ,k_\perp )$ and $\delta _{g}(\epsilon )$ can be found in Refs. [30, 33]. For completeness, we report them in Appendix A, see Eqs. (A.10, A.11, A.12). Finally, $\gamma ^{24,r}_{z,g\rightarrow gg}$ is defined as

4.44 $$\begin{aligned} \gamma ^{24,r}_{z,g\rightarrow gg}= & {} \frac{29}{12}C_A+ C_A\epsilon \left( \frac{371}{24} - \frac{2\pi ^2}{3}\right) \nonumber \\&+C_A\epsilon ^2\left( \frac{1559}{16} - \frac{29\pi ^2}{9}- 24\zeta _3\right) +\mathcal {O}(\epsilon ^3).\nonumber \\ \end{aligned}$$

Note that, as a consequence of spin correlations, the result in Eq. (4.43) contains a finite term $r_\mu r_\nu F_\mathrm{LM}^{\mu \nu }$. This term should be understood as the corresponding matrix element squared where the polarization vector for the gluon $g_3$ is taken to be a particular four-vector $r^\mu$. The precise form of the vector *r* depends on the specific way in which the limit where gluons $g_3$ and $g_4$ become collinear is approached. Since we use the same parametrization of the triple-collinear phase space as in Refs. [30, 33], the explicit form of the vector $r^\mu$ can be taken from these references. As an example, consider the $\omega ^{31,41}$ partition, where $p_{3}$ is written as

4.45 $$\begin{aligned} p_3^\mu = E_3 (1,\sin \theta _{31}\cos \varphi _3,\sin \theta _{31} \sin \varphi _3,\cos \theta _{31}). \end{aligned}$$

Here, $\theta _{31}$ is the relative angle between the momenta of $g_1$ and $g_3$. Upon parametrizing the collinear limit of $g_3$ and $g_4$ as described in Refs. [30, 33], we find the following expression for the vector $r^\mu$

4.46 $$\begin{aligned} r^\mu = (0, -\cos \theta _{31}\cos \varphi _3,-\cos \theta _{31} \sin \varphi _3, \sin \theta _{31}). \end{aligned}$$

Similar to Refs. [30, 33], damping factors with tildes in Eq. (4.43) indicate the damping factors computed in respective collinear limits, e.g.

4.47 $$\begin{aligned} \tilde{\omega }^{31,41}_{4||1} = \lim _{\eta _{41}\rightarrow 0} \omega ^{31,41}. \end{aligned}$$

Finally, the two quantities $\Theta _{ac,bd}$ in Eq. (4.43) are the only entries where the explicit form of the damping factor appears in the fully-unresolved part of the result. They read [30, 33]

4.48 $$\begin{aligned} \Theta _{ac}&= -\left\langle (I-C_{31})\left[ \frac{\eta _{12}}{2\eta _{31}\eta _{32}}\right] \tilde{\omega }^{31,41}_{4||1} \ln \frac{\eta _{31}}{2}\right\rangle + \mathcal {O}(\epsilon ),\nonumber \\ \Theta _{bd}&= - 2\left\langle (I-C_{31})\left[ \frac{\eta _{12}}{2\eta _{31}\eta _{32}}\right] \tilde{\omega }^{31,41}_{3||4}\ln \frac{\eta _{31}}{1-\eta _{31}}\right\rangle + \mathcal {O}(\epsilon ). \end{aligned}$$

Taking the explicit expression for the partition functions shown Appendix A, it is straightforward to obtain

4.49 $$\begin{aligned} \Theta _{ac} = 1+\ln 2+\mathcal {O}(\epsilon ), \,\, \Theta _{bd} = 2-\frac{\pi ^2}{3} + \mathcal {O}(\epsilon ). \end{aligned}$$

#### Soft-regulated triple- and double-collinear contribution

We now discuss the triple- and double-collinear contribution $\left\langle \mathcal {G}^{s_r,c_t}(1,2,3,4)\right\rangle$ shown in Eq. (4.41). As indicated in the previous section, this term includes all the double-unresolved collinear contributions which arise when both gluons $g_{3,4}$ are collinear to either gluon $g_1$ or gluon $g_2$, as well as single-collinear contributions where gluons $g_3$ and $g_4$ are collinear to each other.

This contribution requires a non-trivial integration of the triple-collinear splitting function over energies and angles of particles that participate in the splitting. The relevant computation was performed in Ref. [32]. Using the results presented there, we can write the final result for the soft-regulated triple- and double-collinear contribution as4.50where the first term on the right hand side comes from double-collinear configurations and the second one from the triple-collinear ones. The integral of the triple-collinear splitting function, with soft and collinear singularities subtracted, is denoted by $R^{ggg}$ in Eq. (4.50); it reads [32]

4.51 $$\begin{aligned} R^{ggg}= & {} \frac{C_A^2}{\epsilon }\left( -\frac{1895}{216} +\frac{11\pi ^2}{36}-\frac{11\ln 2}{36}\right. \nonumber \\&\left. + \frac{2\pi ^2\ln 2}{3} +\frac{11\ln ^2 2}{2}-\frac{\zeta _3}{8}\right) \nonumber \\&+C_A^2\bigg (-\frac{335}{8}-\frac{83\pi ^2}{144} + \frac{71\pi ^4}{1440}\nonumber \\&+\frac{845\ln 2}{108}+\frac{187\pi ^2\ln 2}{36}-\frac{169\ln ^2 2}{18}\nonumber \\&-\frac{25\pi ^2\ln ^2 2}{12}-\frac{176\ln ^3 2}{9}-\frac{\ln ^4 2}{12}\nonumber \\&- 2 \text {Li}_4\left( \frac{1}{2}\right) +\frac{121\zeta _3}{8}+\frac{59\ln 2 \zeta _3}{4}\bigg ). \end{aligned}$$

### Real-virtual contribution

We now turn to the discussion of real-virtual contributions. Their calculation is similar to the NLO case discussed in Sect. 3. As in the previous section, we illustrate the most important steps of the real-virtual calculation for the three-gluon final state. Similar to NLO, we introduce a phase-space partitioning and write

4.52 $$\begin{aligned}&\left\langle F_\mathrm{LV}(1_g,2_g,3_g)\right\rangle _\delta \nonumber \\&\quad = 6\left\langle (I-C_{31})\omega ^{31}(I-S_3)\tilde{s}_{12}F_\mathrm{LV}(1_g,2_g,3_g) \right\rangle _\delta \nonumber \\&\qquad + 3\left\langle S_3 \tilde{s}_{12}F_\mathrm{LV}(1_g,2_g,3_g)\right\rangle _\delta \nonumber \\&\qquad +6\left\langle C_{31}(I-S_3)\tilde{s}_{12}F_\mathrm{LV}(1_g,2_g,3_g) \right\rangle _\delta . \end{aligned}$$

We note that the $1\leftrightarrow 2$ symmetry was used to simplify Eq. (4.52). The first term on the right hand side of Eq. (4.52) is fully regulated. The terms on the second line are soft and collinear subtractions, which we now discuss.

The starting point for the calculation of the soft subtraction contribution is the factorization property of the one-loop amplitude [38], that leads to

4.53 $$\begin{aligned}&S_3\bigg [2{\mathfrak {R}}\big [\mathcal {M}^\mathrm{tree}(1_g,2_g,3_g) \mathcal {M}^{\mathrm{1-loop},*}(1_g,2_g,3_g)\big ]\bigg ] \nonumber \\&\quad =\left[ \frac{g_s^2 e^{\epsilon \gamma _E}}{\Gamma (1-\epsilon )}\right] \frac{ 2C_A(p_1\cdot p_2)}{(p_1\cdot p_3)(p_2\cdot p_3)}\nonumber \\&\qquad \times \Bigg \{\bigg [2{\mathfrak {R}}\big [\mathcal {M}^\mathrm{tree}(1_g,2_g) \mathcal {M}^{\mathrm{1-loop},*}(1_g,2_g)\big ]\nonumber \\&\qquad -\frac{\beta _0}{\epsilon }\left( \frac{\alpha _s(\mu )}{2\pi }\right) |\mathcal {M}^\mathrm{tree}(1_g,2_g)|^2\bigg ]\nonumber \\&\qquad -C_A\frac{[\alpha _s]}{\epsilon ^2}\frac{\Gamma ^5(1-\epsilon )\Gamma ^3(1+\epsilon )}{\Gamma ^2(1-2\epsilon )\Gamma (1+2\epsilon )}\nonumber \\&\qquad \times \left( \frac{\eta _{12}}{\eta _{31}\eta _{32}}\right) ^{\epsilon } \left( \frac{4 E_3^2}{\mu ^2}\right) ^{-\epsilon } |\mathcal {M}^\mathrm{tree}(1_g,2_g)|^2\Bigg \}, \end{aligned}$$

with

4.54 $$\begin{aligned} \beta _0 = \frac{11}{6}C_A- \frac{2}{3}T_Rn_f. \end{aligned}$$

The appearance of the $\beta _0$ term in Eq. (4.53) is related to the fact that we work with UV-renormalized amplitudes. Starting from Eq. (4.53), we follow the discussion presented in Sect. 3 and obtain

4.55 $$\begin{aligned}&3\left\langle S_3\tilde{s}_{12}F_\mathrm{LV}(1_g,2_g,3_g)\right\rangle _\delta \nonumber \\&\quad =2C_A\frac{[\alpha _s]}{\epsilon ^2}\left( \frac{\mu ^2}{m_{H}^2}\right) ^{\epsilon } \frac{\Gamma ^2(1-\epsilon )}{\Gamma (1-2\epsilon )}\nonumber \\&\qquad \times \left[ \left\langle F_\mathrm{LV}(1_g,2_g)\right\rangle _\delta -\frac{\beta _0}{\epsilon }\left( \frac{\alpha _s(\mu )}{2\pi }\right) \left\langle F_\mathrm{LM}(1_g,2_g)\right\rangle _\delta \right] \nonumber \\&\qquad -\frac{C_A^2}{2} \frac{[\alpha _s]^2}{\epsilon ^4} \left( \frac{\mu ^2}{m_{H}^2}\right) ^{2\epsilon }\nonumber \\&\qquad \times \frac{\Gamma ^5(1-\epsilon )\Gamma ^3(1+\epsilon )}{\Gamma (1-4\epsilon )\Gamma (1+2\epsilon )} \left\langle F_\mathrm{LM}(1_g,2_g)\right\rangle _\delta . \end{aligned}$$

Next we consider the collinear subtraction. At one-loop, the collinear factorization of one-loop amplitudes leads to [39]

4.56 $$\begin{aligned}&C_{31}\bigg [2{\mathfrak {R}}\big [\mathcal {M}^\mathrm{tree}(1_g,2_g,3_g) \mathcal {M}^{\mathrm{1-loop},*}(1_g,2_g,3_g)\big ]\bigg ] \nonumber \\&\quad =\frac{[g_s^2 e^{\epsilon \gamma _E}/\Gamma (1-\epsilon )]}{p_1\cdot p_3} \times \Bigg \{P_{gg}\left( \frac{E_1}{E_{13}}\right) \nonumber \\&\qquad \otimes \bigg [2{\mathfrak {R}}\big [\mathcal {M}^\mathrm{tree}(13_g,2_g) \mathcal {M}^{\mathrm{1-loop},*}(13_g,2_g)\big ]\nonumber \\&\qquad -\frac{\beta _0}{\epsilon }\left( \frac{\alpha _s(\mu )}{2\pi }\right) |\mathcal {M}^\mathrm{tree}(13_g,2_g)|^2\bigg ]\nonumber \\&\qquad +[\alpha _s]\frac{\Gamma ^3(1-\epsilon ) \Gamma (1+\epsilon )}{\Gamma (1-2\epsilon )}{\mathfrak {R}}\nonumber \\&\qquad \times \left[ \left( -s_{13}\right) ^{-\epsilon } P^{(1)}_{gg} \left( \frac{E_1}{E_{13}}\right) \right] \otimes |\mathcal {M}^\mathrm{tree}(13_g,2_g)|^2\Bigg \}, \end{aligned}$$

where $s_{13} = 2 p_1 \cdot p_3 +i0$. We remind the reader that the notation “$13_g$” indicates a gluon that has the same direction as the gluon $g_1$ but whose energy $E_{13}$ is given by $E_{13}=E_1+E_3$. As in Sect. 3, the symbol $\otimes$ in Eq. (4.56) indicates a contraction of the one-loop spin-correlated splitting function $P_{gg}^{(1)}$ with the relevant scattering amplitudes. The one-loop splitting function $P_{gg}^{(1)}$ was computed in Ref. [39]; we report it in Appendix A for convenience. We note that, at variance with the production case, the splitting function $P_{gg}^{(1)}$ is manifestly real for the decay kinematics. Following the same steps as in the NLO calculation described in Sect. 3, we obtain

4.57 $$\begin{aligned}&6\left\langle C_{31}(I-S_{3}) \tilde{s}_{12}F_\mathrm{LV}(1_g,2_g,3_g)\right\rangle _\delta \nonumber \\&\quad = \frac{[\alpha _s]}{\epsilon }\left( \frac{\mu ^2}{m_{H}^2}\right) ^{\epsilon } \frac{\Gamma ^2(1-\epsilon )}{\Gamma (1-2\epsilon )}\nonumber \\&\qquad \times 2 \gamma _{z,g\rightarrow gg}^{22}\bigg [\left\langle F_\mathrm{LV}(1_g,2_g)\right\rangle _\delta \nonumber \\&\qquad - \frac{\beta _0}{\epsilon }\left( \frac{\alpha _s(\mu )}{2\pi }\bigg ) \left\langle F_\mathrm{LM}(1_g,2_g)\right\rangle _\delta \right] \nonumber \\&\qquad -\frac{[\alpha _s]^2}{\epsilon }\left( \frac{\mu ^2}{m_{H}^2}\right) ^{2\epsilon }\nonumber \\&\qquad \times \frac{\Gamma (1-2\epsilon )\Gamma (1-\epsilon )}{\Gamma (1-3\epsilon )} \gamma _{z,g\rightarrow gg}^\mathrm{1-loop} \left\langle F_\mathrm{LM}(1_g,2_g)\right\rangle _\delta . \end{aligned}$$

In Eq. (4.57), $\gamma _{z,g\rightarrow gg}^\mathrm{1-loop}$ is the one-loop anomalous dimension, analogous to $\gamma _{z,g\rightarrow gg}$, obtained by integrating $P^{(1)}_{gg}$ in Eq. (4.56) over the energy fraction $E_1/E_{13}$. Its explicit expression is reported in Appendix A.

Finally, it is also convenient to explicitly extract the $1/\epsilon$-poles from the $F_\mathrm{LV}$ terms in Eqs. (4.52, 4.55, 4.57). Their structure is well-known [35] and we have already discussed it in Refs. [30, 33] using our notations. For completeness, we report the relevant formulas below

4.58 $$\begin{aligned}&\left\langle F_\mathrm{LV}(1_g,2_g)\right\rangle _\delta \nonumber \\&\quad =-2\cos (\epsilon \pi )[\alpha _s]\left( \frac{\mu ^2}{m_{H}^2}\right) ^{\epsilon } \left[ \frac{C_A}{\epsilon ^2} + \frac{\beta _0}{\epsilon }\right] \nonumber \\&\qquad \times \left\langle F_\mathrm{LM}(1_g,2_g)\right\rangle _\delta +\left\langle F_{\mathrm{LV}}^\mathrm{fin}(1_g,2_g)\right\rangle _\delta \nonumber \\&\left\langle F_\mathrm{LV}(1_g,2_g,3_g)\right\rangle _\delta \nonumber \\&\quad =\left\langle F_{\mathrm{LV}}^\mathrm{fin}(1_g,2_g,3_g)\right\rangle _\delta -\cos (\epsilon \pi )[\alpha _s]\nonumber \\&\qquad \times \left[ \frac{C_A}{\epsilon ^2} + \frac{\beta _0}{\epsilon }\right] \Bigg \langle \left[ \left( \frac{\mu ^2}{s_{12}}\right) ^{\epsilon }\right. \nonumber \\&\left. \qquad +\left( \frac{\mu ^2}{s_{13}}\right) ^{\epsilon } +\left( \frac{\mu ^2}{s_{23}}\right) ^{\epsilon }\right] F_\mathrm{LM}(1_g,2_g,3_g)\Bigg \rangle _\delta . \end{aligned}$$

In Eq. (4.58) the functions $F_{\mathrm{LV}}^\mathrm{fin}$ are finite in four dimensions and $s_{ij} = 2 p_i\cdot p_j >0$.

### Double-virtual corrections

The double-virtual contribution is identical to those in the production case described in Refs. [30, 33]. For convenience, we report the relevant formulas here. Following Ref. [35], we extract all the $\epsilon$-poles from the loop amplitudes and write

4.59 $$\begin{aligned}&\left\langle F_\mathrm{LVV}(1_g,2_g)\right\rangle _\delta \nonumber \\&\quad =\left( \frac{\alpha _s(\mu )}{2\pi }\right) ^2\bigg [ \frac{\tilde{I}_{12}^2(\epsilon )}{2} -\frac{\beta _0}{\epsilon } \tilde{I}_{12}(\epsilon )\nonumber \\&\qquad +\left( \frac{\beta _0}{\epsilon } + K\right) \frac{e^{-\epsilon \gamma _E}\Gamma (1-2\epsilon )}{\Gamma (1-\epsilon )} \tilde{I}_{12}(2\epsilon ) +\frac{H_g}{\epsilon }\bigg ]\nonumber \\&\qquad \times \left\langle F_\mathrm{LM}(1_g,2_g)\right\rangle _\delta +\left( \frac{\alpha _s(\mu )}{2\pi }\right) \tilde{I}_{12}(\epsilon ) \left\langle F_{\mathrm{LV}}^\mathrm{fin}(1_g,2_g)\right\rangle _\delta \nonumber \\&\qquad +\left\langle F_{\mathrm{LVV}}^\mathrm{fin}(1_g,2_g)\right\rangle _\delta +\left\langle F_{\mathrm{LV}^2}^\mathrm{fin}(1_g,2_g)\right\rangle _\delta , \end{aligned}$$

where

4.60 $$\begin{aligned} \tilde{I}_{12}(\epsilon )= & {} -2\cos (\epsilon \pi )\nonumber \\&\times \left[ \frac{e^{\epsilon \gamma _E}}{\Gamma (1-\epsilon )}\right] \left( \frac{\mu ^2}{m_{H}^2}\right) ^{\epsilon } \left[ \frac{C_A}{\epsilon ^2}+\frac{\beta _0}{\epsilon }\right] , \end{aligned}$$

with $\beta _0$ defined in Eq. (4.54) and

4.61 $$\begin{aligned} K= & {} \left( \frac{67}{18}-\frac{\pi ^2}{6}\right) C_A- \frac{10}{9}T_Rn_f,\nonumber \\ H_g= & {} C_A^2 \left( \frac{5}{12} + \frac{11\pi ^2}{144} + \frac{\zeta _3}{2}\right) \nonumber \\&-C_An_f\left( \frac{29}{27}+\frac{\pi ^2}{72}\right) +\frac{C_Fn_f}{2} + \frac{5n_f^2}{27}. \end{aligned}$$

Finally, we note that $\left\langle F_{\mathrm{LV}}^\mathrm{fin}(1_g,2_g)\right\rangle$ is defined in Eq. (3.33), and $\left\langle F_{\mathrm{LVV}}^\mathrm{fin}(1_g,2_g)\right\rangle$, $\left\langle F_{\mathrm{LV}^2}^\mathrm{fin}(1_g,2_g)\right\rangle$ are finite remainders, see Appendix A in Ref. [33] for details.

### Final result

The sum of the different contributions discussed in the previous sections gives a result that is finite in the $\epsilon \rightarrow 0$ limit. Repeating similar calculations for all the other partonic channels, we obtain the full NNLO QCD corrections to the decay $H \rightarrow gg$. We write the result as the sum of contributions with different final state multiplicities, cf. Eq. (2.9)

4.62 $$\begin{aligned} \mathrm{d}\Gamma ^\mathrm{NNLO}= \mathrm{d}\Gamma ^\mathrm{NNLO}_{H\rightarrow 4} +\mathrm{d}\Gamma ^\mathrm{NNLO}_{H\rightarrow 3} + \mathrm{d}\Gamma ^\mathrm{NNLO}_{H\rightarrow 2}. \end{aligned}$$

The contribution of the four-parton final state reads4.63where $F_\mathrm{LM}(1,2,3,4)$ is defined in Eq. (4.20). Similarly, the three-parton contribution reads

4.64 $$\begin{aligned} \mathrm{d}\Gamma ^{\mathrm{NNLO}}_{H\rightarrow 3}&=\bigg \langle \hat{\mathcal {O}}_\mathrm{NLO}\mathcal {J}_{ggg} \big [3 \tilde{s}_{12}F_\mathrm{LM}(1_g,2_g,3_g)\big ]\nonumber \\&\quad +n_f\big [\hat{\mathcal {O}}_\mathrm{NLO}\mathcal {J}_{gqq}\; \tilde{s}_{12}F_\mathrm{LM}(1_g,2_q,3_{\bar{q}})\nonumber \\&\quad +\hat{\mathcal {O}}_\mathrm{NLO}\mathcal {J}_{qgq}\; \tilde{s}_{12}F_\mathrm{LM}(1_q,2_g,3_{\bar{q}})\nonumber \\&\quad +\hat{\mathcal {O}}_\mathrm{NLO}\mathcal {J}_{qqg}\; \tilde{s}_{12}F_\mathrm{LM}(1_q,2_{\bar{q}},3_g)\big ] \bigg \rangle _\delta \nonumber \\&\quad +\gamma _{k_\perp ,g}\left\langle \hat{\mathcal {O}}_\mathrm{NLO}\tilde{s}_{12}r_\mu r_\nu \right. \nonumber \\&\qquad \left. \times \left[ 3 F_\mathrm{LM}^{\mu \nu }(1_g,2_g,3_g) +n_fF_\mathrm{LM}^{\mu \nu }(1_q,2_{\bar{q}},3_g)\right] \right\rangle _\delta , \end{aligned}$$

where

4.65 $$\begin{aligned} \hat{\mathcal {O}}_\mathrm{NLO}= (I - S_3)(I - C_{31} - C_{32})\left( \omega ^{31}+\omega ^{32}\right) , \end{aligned}$$

and

4.66 $$\begin{aligned} \mathcal {J}_{ijk} = \mathcal {J}_{ijk}^{(1)} + \mathcal {J}_{ijk}^{(2)}. \end{aligned}$$

The functions $\mathcal {J}^{(1,2)}$ are defined as

4.67 $$\begin{aligned} \mathcal {J}_{ggg}^{(1)}&= C_A\left[ \widetilde{\mathcal {K}}_{12} + \widetilde{\mathcal {K}}_{13} + \widetilde{\mathcal {K}}_{23}\right] +\beta _0 \ln (\eta _{12}\eta _{13}\eta _{23}),\nonumber \\ \mathcal {J}_{gqq}^{(1)}&= C_A\left[ \widetilde{\mathcal {K}}_{12}+\widetilde{\mathcal {K}}_{13}\right] \nonumber \\&\quad +(2C_F-C_A) \widetilde{\mathcal {K}}_{23}+ (2C_F-C_A)\frac{3}{2} \ln (\eta _{23})\nonumber \\&\quad + \frac{\beta _0}{2}\ln \left( \frac{E_2 E_3 \eta _{12}\eta _{13}}{E_1^2}\right) + \frac{3}{4}C_A\ln \left( \frac{E_1^2\eta _{12}\eta _{13}}{E_2 E_3}\right) ,\nonumber \\ \mathcal {J}_{qgq}^{(1)}&= C_A\left[ \widetilde{\mathcal {K}}_{12}+\widetilde{\mathcal {K}}_{23}\right] \nonumber \\&\quad +(2C_F-C_A) \widetilde{\mathcal {K}}_{13}+ (2C_F-C_A)\frac{3}{2} \ln (\eta _{13})\nonumber \\&\quad + \frac{\beta _0}{2}\ln \left( \frac{E_1 E_3 \eta _{12}\eta _{23}}{E_2^2}\right) + \frac{3}{4}C_A\ln \left( \frac{E_2^2\eta _{12}\eta _{23}}{E_1 E_3}\right) ,\nonumber \\ \mathcal {J}_{qqg}^{(1)}&= C_A\left[ \widetilde{\mathcal {K}}_{13}+\widetilde{\mathcal {K}}_{23}\right] \nonumber \\&\quad +(2C_F-C_A) \widetilde{\mathcal {K}}_{12}+ (2C_F-C_A)\frac{3}{2} \ln (\eta _{12})\nonumber \\&\quad + \frac{\beta _0}{2}\ln \left( \frac{E_1 E_2 \eta _{13}\eta _{23}}{E_3^2}\right) + \frac{3}{4}C_A\ln \left( \frac{E_3^2\eta _{13}\eta _{23}}{E_1 E_2}\right) , \end{aligned}$$

and

4.68 $$\begin{aligned} \mathcal {J}^{(2)}_{ijk}= & {} \gamma '_i + \gamma '_j + \tilde{\gamma }'_k -\tilde{\omega }^{31,41}_{4||1}\ln \left( \frac{\eta _{13}}{2}\right) \nonumber \\&\quad \times \left( \gamma _i + 2 C_i \ln \frac{E_3}{E_1}\right) -\tilde{\omega }^{32,42}_{4||2}\ln \left( \frac{\eta _{23}}{2}\right) \nonumber \\&\quad \times \left( \gamma _j + 2 C_j \ln \frac{E_3}{E_2}\right) -\left[ \tilde{\omega }^{31,41}_{3||4} \ln \left( \frac{\eta _{13}}{4(1-\eta _{13})}\right) \right. \nonumber \\&\qquad \left. +\tilde{\omega }^{32,42}_{3||4} \ln \left( \frac{\eta _{23}}{4(1-\eta _{23})}\right) \right] \gamma _k, \end{aligned}$$

where $C_q=C_F$ and $C_g = C_A$. The various constants and functions used in Eqs. (4.67, 4.68) can be found in Appendix A.

Finally, the two-parton contribution reads

4.69 $$\begin{aligned}&\mathrm{d}\Gamma ^\mathrm{NNLO}_{H\rightarrow 2} \nonumber \\&\quad =\left( \frac{\alpha _s(\mu )}{2\pi }\right) ^2 \left\langle F_\mathrm{LM}(1_g,2_g)\right\rangle _\delta \nonumber \\&\qquad \times \Bigg \{C_A^2\bigg [\frac{65837}{324} -\frac{203\pi ^2}{12}+\frac{469\pi ^4}{720}\nonumber \\&\qquad + \ln ^2 2\left( \frac{\pi ^2}{6}-2\right) -\frac{\ln ^4 2}{6}-4\text {Li}_4\left( \frac{1}{2}\right) \nonumber \\&\qquad +\ln 2\left( 3 + \frac{11\pi ^2}{9} - \frac{7\zeta _3}{2}\right) -\frac{1859\zeta _3}{36}\nonumber \\&\qquad +\ln \left( \frac{\mu ^2}{m_{H}^2}\right) \left( \frac{1429}{54}-\frac{11\pi ^2}{8}-\zeta _3\right) +\frac{203}{18}\Theta _{ac}\nonumber \\&\qquad +\left( \frac{11\ln 2}{3}+\frac{\pi ^2}{3} -\frac{131}{36}\right) \Theta _{bd}\bigg ]\nonumber \\&\qquad +C_An_f\bigg [-\frac{5701}{81} + \frac{673\pi ^2}{216}\nonumber \\&\qquad -\ln 2\left( 3 + \frac{2\pi ^2}{9}\right) +2\ln ^2 2+\frac{49\zeta _3}{18}\nonumber \\&\qquad +\ln \left( \frac{\mu ^2}{m_{H}^2}\right) \left( \frac{\pi ^2}{4}-\frac{15}{2}\right) \nonumber \\&\qquad -\frac{41}{18}\Theta _{ac} + \left( \frac{23}{36} -\frac{2\ln 2}{3}\right) \Theta _{bd}\bigg ]\nonumber \\&\qquad +C_Fn_f\bigg [\frac{-27}{4} + \frac{\pi ^2}{6}+\frac{20\zeta _3}{3} -\ln \left( \frac{\mu ^2}{m_{H}^2}\right) \bigg ]\nonumber \\&\qquad +n_f^2\bigg [\frac{1889}{324}-\frac{5\pi ^2}{108} + \frac{13}{27}\ln \left( \frac{\mu ^2}{m_{H}^2}\right) \bigg ] \Bigg \} \nonumber \\&\qquad + \left( \frac{\alpha _s(\mu )}{2\pi }\right) \left[ 2\gamma '_g +\frac{2\pi ^2}{3}C_A\right] \left\langle F_{\mathrm{LV}}^\mathrm{fin}(1_g,2_g)\right\rangle _\delta . \end{aligned}$$

where $\Theta _{ij}$ depends on the choice of the partition functions and are given in Eqs. (4.48, 4.49).

## Higgs decay to $b\bar{b}$

In this section, we consider the second type of decays, $H \rightarrow b \bar{b}$.^8^ The calculation of NLO and NNLO corrections proceeds along the same lines as before but is significantly simpler. For this reason, we do not discuss it and just report the results of the calculation. Although we consider the $H\rightarrow b\bar{b}$ process for definiteness, we stress that the formulas presented in this section can be applied verbatim to other decays of color singlets to quarks, e.g. $V\rightarrow q\bar{q}'$, $V = Z,W$.

The NLO computation in this case is simpler than for the $H\rightarrow gg$ process discussed in Sect. 3 because, when the Higgs boson decays into a $b \bar{b} g$ final state, singularities only arise when the gluon becomes soft and/or collinear to one of the *b* quarks; in other words, the *b* quarks must be hard. For this reason, there is no need to introduce the $\tilde{s}_{ij}$-partitioning. Repeating the NLO QCD calculation described in Sect. 3, we then obtain

5.1 $$\begin{aligned} \mathrm{d}\Gamma ^\mathrm{NLO}_{H\rightarrow 2}&= \frac{\alpha _s(\mu )}{2\pi }\left( 2\gamma '_q + \frac{2\pi ^2}{3}C_F\right) \nonumber \\&\quad \times \left\langle F_\mathrm{LM}(1_b,2_{\bar{b}})\right\rangle _\delta + \left\langle F_{\mathrm{LV}}^\mathrm{fin}(1_b,2_{\bar{b}})\right\rangle _\delta ,\nonumber \\ \mathrm{d}\Gamma ^\mathrm{NLO}_{H\rightarrow 3}&= \left\langle \hat{\mathcal {O}}_\mathrm{NLO}F_\mathrm{LM}(1_b,2_{\bar{b}},3_g)\right\rangle _\delta , \end{aligned}$$

where $\gamma '_q$ is given in Eq. (A.8) and $F_{\mathrm{LV}}^\mathrm{fin}(1_b,2_{\bar{b}})$ is a finite virtual remainder analogous to $F_\mathrm{LV}(1_g,2_g)$ in Eq. (3.33), see Appendix A in Ref. [33] for its explicit definition.

At NNLO, we also do not require any additional partitioning except perhaps for the 4*b* final state that arises from the prompt decay of the Higgs boson.^9^ We show in Appendix C that the contribution of this subprocess to the decay rate can be written as a sum of two terms: a term that coincides with the contribution of the decay $H \rightarrow b \bar{b} q \bar{q}$, $q\ne b$, where only *b* and $\bar{b}$ can be prompt and the $q \bar{q}$ pair originates from gluon splitting, and an interference term. The first term can be treated without any partitioning since the hard partons are always the two *b*-quarks. The interference term has only a triple-collinear singularity that maps onto the corresponding splitting function. Its proper treatment is described in Appendix C.

The NNLO contribution to $H \rightarrow b \bar{b}$ decay is then computed following the steps discussed in the previous section. We write the NNLO contribution as a sum of “fixed-multiplicity” terms $\mathrm{d}\Gamma ^\mathrm{NNLO}_{Q\rightarrow i}$, $i =2,3,4$. The four-parton contribution reads5.2where now

5.3 $$\begin{aligned}&\mathcal {F}(1,2,3,4) \nonumber \\&\quad =\left[ F_\mathrm{LM}(1_b,2_{\bar{b}},3_g,4_g) +n_fF_\mathrm{LM}(1_b,2_{\bar{b}},3_q,4_{\bar{q}}) \right. \nonumber \\&\qquad \left. +F_\mathrm{LM}^\mathrm{int}(1,2,3,4)\right] \theta (E_3-E_4), \end{aligned}$$

with $F_\mathrm{LM}^\mathrm{int}$ defined in Appendix C. The three-parton contribution reads

5.4 $$\begin{aligned}&\mathrm{d}\Gamma ^\mathrm{NNLO}_{H\rightarrow 3} \nonumber \\&\quad = \frac{\alpha _s(\mu )}{2\pi }\left[ \left\langle \hat{\mathcal {O}}_\mathrm{NLO}\mathcal {J}_{ qqg} F_\mathrm{LM}(1_b,2_{\bar{b}},3_g)\right. \right. \nonumber \\&\qquad \left. \left. +\gamma _{k_\perp ,g}\hat{\mathcal {O}}_\mathrm{NLO}r_\mu r_\nu F_\mathrm{LM}^{\mu \nu }(1_b,2_{\bar{b}},3_g) \right\rangle _\delta \right] , \end{aligned}$$

where the function $\mathcal {J}_{qqg}$ is defined in Eq. (4.66) and $\gamma _{k_\perp ,g}$ can be found in Appendix A.

Finally, the two-parton contribution reads

5.5 $$\begin{aligned}&\mathrm{d}\Gamma ^{\mathrm{NNLO}}_{H\rightarrow 2} \nonumber \\&\quad =\left( \frac{\alpha _s(\mu )}{2\pi }\right) \left( 2\gamma '_q + \frac{2\pi ^2}{3}C_F\right) \left\langle F_{\mathrm{LV}}^\mathrm{fin}(1_b,2_{\bar{b}})\right\rangle _\delta \nonumber \\&\qquad +\left\langle F_{\mathrm{LVV}}^\mathrm{fin}(1_b,2_{\bar{b}})\right\rangle _\delta +\left\langle F_{\mathrm{LV}^2}^\mathrm{fin}(1_b,2_{\bar{b}})\right\rangle _\delta \nonumber \\&\qquad +\left( \frac{\alpha _s(\mu )}{2\pi }\right) ^2 \left\langle F_\mathrm{LM}(1_b,2_{\bar{b}})\right\rangle \nonumber \\&\qquad \times \Bigg \{C_F^2\bigg [\frac{1081}{16} -\frac{67\pi ^2}{6} +\frac{2\pi ^4}{3}+9\zeta _3 + \ln \left( \frac{\mu ^2}{m_{H}^2}\right) \nonumber \\&\qquad \times \left( \frac{3}{4}-\pi ^2+12\zeta _3\right) + 7 \Theta _{ac} \bigg ]\nonumber \\&\qquad +C_AC_F\bigg [\frac{115441}{1296}-\frac{29\pi ^2}{8}-\frac{11\pi ^4}{720} +\ln ^2 2\left( \frac{\pi ^2}{6}-2\right) \nonumber \\&\qquad -\frac{\ln ^4 2}{6}+ \ln 2\left( \frac{8}{3} +\frac{11\pi ^2}{9}-\frac{7\zeta _3}{2}\right) -\frac{2135\zeta _3}{36}-4\text {Li}_4\left( \frac{1}{2}\right) \nonumber \\&\qquad +\ln \left( \frac{\mu ^2}{m_{H}^2}\right) \left( \frac{2329}{108}-\frac{19\pi ^2}{72}-13\zeta _3\right) \nonumber \\&\qquad +\left( \frac{11\ln 2}{3}+\frac{\pi ^2}{3} -\frac{131}{36}\right) \Theta _{bd}\bigg ]\nonumber \\&\qquad +C_Fn_f\bigg [-\frac{9929}{648} + \frac{5\pi ^2}{9} -\ln 2\left( \frac{8}{3}+\frac{2\pi ^2}{9}\right) \nonumber \\&\qquad +2\ln ^2 2 + \frac{145\zeta _3}{18} +\ln \left( \frac{\mu ^2}{m_{H}^2}\right) \left( -\frac{209}{54}+\frac{5\pi ^2}{36}\right) \nonumber \\&\qquad +\left( \frac{23}{36}-\frac{2\ln 2}{3}\right) \Theta _{bd} \bigg ]\Bigg \}, \end{aligned}$$

where $\Theta _{ij}$ are defined in Eqs. (4.48, 4.49).

## Validation of results

In this section, we use the analytic formulas for the fully-differential decay rates presented above to calculate the NNLO QCD corrections to decays $H \rightarrow gg$ and $H \rightarrow b \bar{b}$.^10^ We compare these results with analytic formulas extracted from Refs. [42–44] to validate our calculations.^11^

We begin with the decay process $H \rightarrow gg$, which was discussed in Sect. 4. We consider a Higgs boson of mass $m_{H}= 125$ GeV which couples to gluons through the effective Lagrangian

6.1 $$\begin{aligned} \mathcal {L}_{ Hgg} = -\lambda _{Hgg} H G_{\mu \nu }^{(a)} G^{\mu \nu ,(a)}, \end{aligned}$$

where in the $\overline{\mathrm{MS}}$ scheme

6.2 $$\begin{aligned}&\lambda _{Hgg} \nonumber \\&\quad = -\frac{\alpha _s}{12 \pi v} \bigg \{ 1 + \left[ \frac{5}{2}C_A- \frac{3}{2}C_F\right] \left( \frac{\alpha _s}{2\pi }\right) \nonumber \\&\qquad +\bigg [\frac{1063}{144}C_A^2 - \frac{25}{3}C_AC_F+ \frac{27}{8}C_F^2\nonumber \\&\qquad -\frac{47}{72}C_An_f- \frac{5}{8}C_Fn_f-\frac{5}{48}C_A- \frac{C_F}{6}\nonumber \\&\qquad +\ln \left( \frac{\mu ^2}{m_t^2}\right) \bigg (\frac{7}{4}C_A^2 - \frac{11}{4} C_AC_F+ C_Fn_f\bigg )\bigg ]\nonumber \\&\qquad \times \left( \frac{\alpha _s}{2\pi }\right) ^2 + \mathcal {O}(\alpha _s^3)\bigg \}, \end{aligned}$$

with $\alpha _s= \alpha _s(\mu )$ being the renormalized coupling in a theory with 5 massless flavors and *v* is the Higgs vacuum expectation value, see e.g. Ref. [65]. For the numerical results presented below, we use $m_t=173.2$ GeV.

For numerical checks, we split the width for the $H \rightarrow gg$ decay into different color factors, which allows us to check different partonic channels separately. We write

6.3 $$\begin{aligned}&\Gamma (H \rightarrow gg) \nonumber \\&\quad =\Gamma _\mathrm{LO}(H \rightarrow gg) + \Gamma _\mathrm{NLO}(H \rightarrow gg)\nonumber \\&\qquad + \Gamma _\mathrm{NNLO}(H \rightarrow gg) +\mathcal {O}(\alpha _s^5) \nonumber \\&\quad =\Gamma _\mathrm{LO}(H\rightarrow gg) \times \biggl [1 +\left( \frac{\alpha _s}{2\pi }\right) \left( C_AR^{(1)}_{C_A}+n_fR^{(1)}_{n_f} \right) \nonumber \\&\qquad +\left( \frac{\alpha _s}{2\pi }\right) ^2 \left( C_AR^{(2)}_{C_A}+n_fR^{(2)}_{n_f}+n_f^2 R^{(2)}_{n_f^2}\right) \biggr ] + \mathcal {O}(\alpha _s^5), \end{aligned}$$

where the LO decay width that has been factored out is given by $\Gamma _\mathrm{LO}(H \rightarrow gg) = (\alpha _s(\mu ))^2/(72 \pi ^3 v^2)$. The comparison between our results for the NLO and NNLO coefficients $R^{(1,2)}$ and those presented in Ref. [42] is given in Table 1. We present numerical results for a scale $\mu =2 m_H$, in order to avoid accidental cancellations between the renormalization scale $\mu$ and the Higgs mass $m_{H}$ that happen for $\mu =m_{H}$. We observe agreement well below the per mille level for all coefficients.

**Table 1:** Comparison between numerical and analytic results for NLO and NNLO color coefficients appearing in $H \rightarrow gg$ decay. The residual Monte Carlo integration error is given in parentheses. See text for details

Color structure	Numerical result	Analytic result
$R^{(1)}_{C_A}$	62.749 (3)	62.749
$R^{(1)}_{n_f}$	$-$3.2575 (2)	$-$3.2575
$R^{(2)}_{C_A}$	2806.2 (4)	2806.2
$R^{(2)}_{n_f}$	$-$339.63 (1)	$-$339.63
$R^{(2)}_{n_f^2}$	7.4824 (1)	7.4824

We turn now to the decay $H \rightarrow b\bar{b}$. Again, we consider a 125 GeV Higgs boson and five flavors of massless quarks, which allows us to use the results presented in Sect. 5. The Higgs couples to bottom quarks only, through a Yukawa interaction

6.4 $$\begin{aligned} \mathcal {L}_{Hb\bar{b}} = - \frac{y_b}{\sqrt{2}} H b\bar{b}. \end{aligned}$$

Once again, we write the result for the Higgs decay width in terms of different color structures, factoring out the LO decay width $\Gamma _\mathrm{LO}(H\rightarrow b\bar{b}) = 3 y_b^2 m_H /(16\pi )$,

6.5 $$\begin{aligned}&\Gamma (H \rightarrow b\bar{b}) \nonumber \\&\quad =\Gamma _\mathrm{LO}(H \rightarrow b\bar{b}) + \Gamma _\mathrm{NLO}(H \rightarrow b\bar{b}) \nonumber \\&\qquad + \Gamma _\mathrm{NNLO}(H \rightarrow b\bar{b}) + \mathcal {O}(\alpha _s^3) \nonumber \\&\quad = \Gamma _\mathrm{LO}(H\rightarrow b\bar{b}) \times \biggl [1 +\left( \frac{\alpha _s}{2\pi }\right) \left( C_FS^{(1)}_{C_F}\right) \nonumber \\&\qquad +\left( \frac{\alpha _s}{2\pi }\right) ^2 \left( C_F^2 S^{(2)}_{C_F^2}+C_AC_FS^{(2)}_{C_AC_F}\right. \nonumber \\&\qquad \left. + T_RC_Fn_fS^{(2)}_{C_Fn_f} \right) \biggr ] + \mathcal {O}(\alpha _s^3). \end{aligned}$$

The comparison between the coefficients $S^{(1,2)}$ obtained from our numerical code and from the analytic formulas of Ref. [44] are displayed in Table 2. Again, we use the scale $\mu _R = 2m_{H}$ for this comparison. The agreement is consistently below the per mille level across all color structures.

**Table 2:** Comparison between numerical and analytic results for NLO and NNLO color coefficients appearing in $H \rightarrow b\bar{b}$ decay. The residual Monte Carlo integration error is given in parentheses. See text for details

Color structure	Numerical result	Analytic result
$S^{(1)}_{C_F}$	12.659 (2)	12.659
$S^{(2)}_{C_F^2}$	62.59 (1)	62.60
$S^{(2)}_{C_AC_F}$	66.23 (1)	66.23
$S^{(2)}_{C_Fn_f}$	$-$20.24 (1)	$-$20.24

Finally, we compare exclusive jet rates for the $H \rightarrow b\bar{b}$ decay with those reported in Ref. [44]. To do so, we use the JADE clustering algorithm with $y_\mathrm{cut}=0.01$ and the distance measure defined as $y_{ij}=(p_i+p_j)^2$, and choose the scale $\mu =m_{H}$. We obtain

6.6 $$\begin{aligned} \Gamma _{2j}(H \rightarrow b\bar{b})&= \Gamma _\mathrm{LO}(H \rightarrow b\bar{b}) \nonumber \\&\quad \times \left[ 1-27.176(3) \left( \frac{\alpha _s}{2\pi }\right) \right. \nonumber \\&\quad \left. -1240.8(1) \left( \frac{\alpha _s}{2\pi } \right) ^2 \right] +\mathcal {O}(\alpha _s^3)\nonumber \\ \Gamma _{3j}(H \rightarrow b\bar{b})&= \Gamma _\mathrm{LO}(H \rightarrow b\bar{b}) \nonumber \\&\quad \times \left[ 38.509(3) \left( \frac{\alpha _s}{2\pi }\right) \right. \nonumber \\&\quad \left. + 980.6(1) \left( \frac{\alpha _s}{2\pi }\right) ^2 \right] +\mathcal {O}(\alpha _s^3) \nonumber \\ \Gamma _{4j}(H \rightarrow b\bar{b})&= \Gamma _\mathrm{LO}(H \rightarrow b\bar{b})\nonumber \\&\quad \times 376.785(8) \left( \frac{\alpha _s}{2\pi }\right) ^2 + \mathcal {O}(\alpha _s^3). \end{aligned}$$

We note that our results differ from those of Ref. [44] by 1–2%, which is consistent with the errors reported in that reference. The sum of the jet rates gives the total decay rate at the scale $\mu =m_{H}$6.7in excellent agreement with the analytic results at this scale6.8Clearly, the level of numerical precision achieved for the NNLO coefficients in our calculation is excessive since for phenomenological applications it is enough to know widths with sub-percent accuracy. We note that to achieve this level of numerical precision within our framework, one would typically require up to one CPU hour of computation time.

## Conclusion

We presented analytic formulas that describe fully-differential decays of color-singlet particles to $q \bar{q}$ and *gg* final states through NNLO QCD. The results are obtained within the nested soft-collinear subtracted scheme that we proposed earlier in Ref. [30]. The results are remarkably compact and simple to implement in a numerical code. We have validated these results by computing the NNLO QCD corrections to the $H \rightarrow gg$ and $H \rightarrow b \bar{b}$ decay rates and comparing them to independent numerical and analytic computations finding per mille level agreement for observables that are known analytically. In addition to their phenomenological relevance for decays of the Higgs boson and electroweak vector bosons, such as $H \rightarrow gg, H \rightarrow b \bar{b}$ and $V \rightarrow q \bar{q}$, these results provide an important building block for the extension of the nested soft-collinear subtraction scheme which will make it applicable for computations of NNLO QCD corrections to arbitrary processes at hadron colliders.

## Figures and Tables

**Fig. 1:**
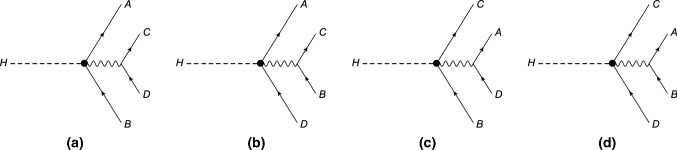
Diagrams for $H \rightarrow b\bar{b}b\bar{b}$ decay. The gluon emission off the bubble in $Hb \bar{b}$ vertex describes diagrams where the gluon is emitted from one of the outgoing quark lines

**Fig. 2:**
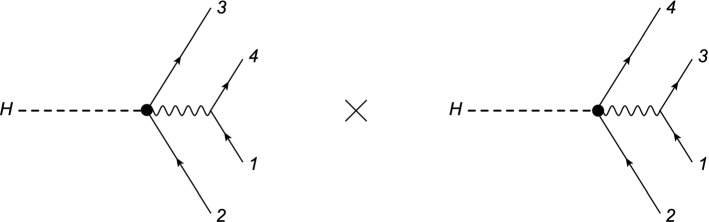
Interference contributions for the triple collinear limit 1||3||4

## Data Availability

This manuscript has no associated data or the data will not be deposited. [Authors’ comment: This is a theoretical article. All the scientific information is contained in analytic formulas reported in the text.]

## Appendix A: Auxiliary quantities

In this appendix, results for various quantities used in this paper are summarized. We start with discussing the partition functions. They read

A.1 $$\begin{aligned} w^{31,41}= & {} \frac{\eta _{32} \eta _{42}}{d_3 d_4 } \left( 1 + \frac{\eta _{31}}{d_{3421}} +\frac{\eta _{41}}{d_{3412}} \right) , \end{aligned}$$

A.2 $$\begin{aligned} w^{32,42}= & {} \frac{\eta _{31} \eta _{41}}{d_3 d_4 } \left( 1+ \frac{\eta _{42}}{d_{3421}} + \frac{\eta _{32}}{d_{3412}}\right) , \end{aligned}$$

A.3 $$\begin{aligned} w^{31,42}= & {} \frac{\eta _{32} \eta _{41} \eta _{43}}{d_3 d_4 d_{3412} }, \;\;\; w^{32,41} = \frac{\eta _{31} \eta _{42} \eta _{43}}{d_3 d_4 d_{3421} }, \end{aligned}$$

where

A.4 $$\begin{aligned}&d_{i=3,4} = \eta _{i1} + \eta _{i2},\;\;\;\; d_{3421} = \eta _{43} + \eta _{32} + \eta _{41},\nonumber \\&d_{3412} = \eta _{43} + \eta _{31} + \eta _{42}, \end{aligned}$$

and

A.5 $$\begin{aligned} \eta _{ij}=(1 - \cos \theta _{ij})/2. \end{aligned}$$

It is straightforward to check that these functions provide a partition of unity

A.6 $$\begin{aligned} w^{31,41} + w^{32,42} + w^{31,42} + w^{32,41} = 1. \end{aligned}$$

We now present formulas for the various anomalous dimensions used in the main text. In our NLO discussion, we used

A.7 $$\begin{aligned}&\gamma _g = \beta _0 = \frac{11}{6}C_A-\frac{2}{3}T_Rn_f,\nonumber \\&\gamma '_g = C_A\left( \frac{67}{9}-\frac{2\pi ^2}{3}\right) - \frac{23}{9}T_Rn_f, \end{aligned}$$

and

A.8 $$\begin{aligned} \gamma _q = \frac{3}{2}C_F, ~~~~ \gamma '_q = C_F\left( \frac{13}{2}-\frac{2\pi ^2}{3}\right) . \end{aligned}$$

At NNLO, we also introduced

A.9 $$\begin{aligned} \gamma _{k_\perp ,g}&= -\frac{C_A}{3}+\frac{2}{3}T_Rn_f,\nonumber \\ \tilde{\gamma }'_g&= C_A\left( \frac{137}{18}-\frac{2\pi ^2}{3}\right) \nonumber \\&\quad - \frac{26}{9}T_Rn_f, ~~~~ \tilde{\gamma }'_q = \gamma '_q. \end{aligned}$$

Following [30, 33], we defined

A.10 $$\begin{aligned} \tilde{\gamma }_g(\epsilon )= & {} \bigg [\frac{11}{6}C_A- \frac{2}{3}T_Rn_f\bigg ]\nonumber \\&+\epsilon \bigg [\left( \frac{137}{18} -\frac{2\pi ^2}{3}\right) C_A- \frac{26}{9} T_Rn_f\bigg ]\nonumber \\&+\epsilon ^2\bigg [\left( \frac{823}{27} -\frac{11\pi ^2}{18} -16 \zeta _3\right) C_A\nonumber \\&+ \left( \frac{2\pi ^2}{9} - \frac{320}{27}\right) T_Rn_f\bigg ] + \mathcal {O}(\epsilon ^3); \end{aligned}$$

A.11 $$\begin{aligned} \tilde{\gamma }_g(\epsilon ,k_\perp )= & {} \bigg [-\frac{C_A}{3} + \frac{2}{3} T_Rn_f\bigg ]\nonumber \\&+ \epsilon \bigg [-\frac{7}{9} C_A+ \frac{20}{9} T_Rn_f\bigg ] + \mathcal {O}(\epsilon ^2); \end{aligned}$$

A.12 $$\begin{aligned} \delta _g(\epsilon )= & {} \bigg [\left( -\frac{131}{72} +\frac{\pi ^2}{6}+\frac{11}{6} \ln (2)\right) C_A\nonumber \\&+\left( \frac{23}{36}-\frac{2}{3} \ln (2)\right) T_Rn_f\bigg ]\nonumber \\&+\epsilon \bigg [\left( -\frac{1541}{216} + \frac{11\pi ^2}{18} - \frac{\ln (2)}{6} + 4 \zeta _3\right) C_A\nonumber \\&+\left( \frac{103}{54} -\frac{2\pi ^2}{9} + \frac{2}{3} \ln (2)\right) T_Rn_f\bigg ] +\nonumber \\&+\epsilon ^2\bigg [\left( -\frac{9607}{324} + \frac{125\pi ^2}{216} + \frac{7\pi ^4}{45} + \ln (2) \right. \nonumber \\&\left. +\frac{11\pi ^2}{18}\ln (2) + \frac{77}{6} \zeta _3\right) C_A\nonumber \\&+ \left( \frac{746}{81} - \frac{5\pi ^2}{108} - \frac{4}{3} \ln (2)\right. \nonumber \\&\left. - \frac{2\pi ^2}{9}\ln (2) - \frac{14}{3} \zeta _3\right) T_Rn_f\bigg ]. \end{aligned}$$

In the “gluon-only” case, discussed in Sect. 4, one should set $n_f=0$ in the above formulas.

We now discuss the one-loop gluon splitting function. It reads [39]

A.13 $$\begin{aligned} P^{(1),{\mu \nu }}_{gg}(z)= & {} \frac{C_A}{\epsilon ^2}\Bigg \{z^{\epsilon }F_{21}(\epsilon ,\epsilon ,1+\epsilon ,1-z)\nonumber \\&+(1-z)^{\epsilon }F_{21}(\epsilon ,\epsilon ,1+\epsilon ,z)\nonumber \\&-\Gamma (1+\epsilon )\Gamma (1-\epsilon ) \left[ \left( \frac{z}{1-z}\right) ^{\epsilon }\right. \nonumber \\&\left. +\left( \frac{1-z}{z}\right) ^{\epsilon } \right] -1\Bigg \}P^{\mu \nu }_{gg}(z)\nonumber \\&+\frac{n_f-C_A(1-\epsilon )}{(1-\epsilon )(1-2\epsilon )(3-2\epsilon )} P^{\mu \nu ,\mathrm{new}}_{gg}(z).\nonumber \\ \end{aligned}$$

Here, $F_{21}$ is the hypergeometric function. We note that the result for the splitting function Eq. (A.13) is written in the conventional dimensional regularization scheme (CDR).

The splitting functions $P^{\mu \nu }_{gg}$, $P^{\mu \nu ,\mathrm{new}}_{gg}$ read

A.14

with $\kappa _\perp = k_\perp /\sqrt{-k_\perp ^2}$. The transversal metric tensor $g_\perp ^{\mu \nu }$ and the transversal vector $k_\perp$ are defined relative to the four-momentum of the collinear gluon, in the standard way [38]. The $d-$dimensional spin averages of the splitting functions give

A.15

We use these results to construct the spin-averaged splitting function $P^{(\mathrm 1)}_{gg}$; we then integrate it over *z* to obtain the anomalous dimension $\gamma ^\mathrm{1-loop}_{z,g\rightarrow gg}$ following a similar procedure to the one described for the tree-level splitting function, see the discussion leading to Eq. (3.24). We find

A.16 $$\begin{aligned} \gamma ^\mathrm{1-loop}_{z,g\rightarrow gg}= & {} \frac{11}{6}\frac{C_A^2}{\epsilon ^2} +\frac{C_A^2}{\epsilon }\left( \frac{134}{9}-\frac{4\pi ^2}{3}\right) \nonumber \\&+C_A^2\left( \frac{1013}{9}-\frac{44\pi ^2}{9}-60\zeta _3\right) -\frac{C_An_f}{6}\nonumber \\&+\epsilon \bigg [C_A^2\left( \frac{14635}{18} -\frac{335\pi ^2}{9}-\frac{106\pi ^4}{45} -\frac{550}{3}\zeta _3\right) \nonumber \\&-\frac{31}{18}C_An_f\bigg ]+\mathcal {O}(\epsilon ^2). \end{aligned}$$

Finally, the function $\widetilde{\mathcal {K}}_{ij}$ reads

A.17 $$\begin{aligned} \widetilde{\mathcal {K}}_{ij}= & {} \text {Li}_2(1-\eta _{ij})+\frac{\ln ^2(E_i/E_j)}{2}\nonumber \\&- \ln \left( \frac{E_i E_j}{E_3^2}\right) \ln (\eta _{ij})+ \frac{\pi ^2}{3}. \end{aligned}$$

## Appendix B: Double-collinear phase space

In this appendix we describe the parametrization of the double-collinear phase space, which turns out to be somewhat convoluted in this case.^12^

We consider the phase space integral

B.1 $$\begin{aligned} I= & {} \int [df_{1}][df_{2}][df_{3}][df_{4}] (2\pi )^d \delta ^{(d)}\nonumber \\&\times (p_H-p_1-p_2-p_3-p_4), \end{aligned}$$

with $p_H$ at rest, $p_H=(m_{H},\vec {0})$. Our goal is to write the integration measure in Eq. (B.1) in such a way that the energies $E_{3,4}$, the relative angle $\theta _{13}$ between $p_1$ and $p_3$ and the relative angle between $p_2$ and $p_4$, $\theta _{24}$, are used as the integration variables. We first integrate over $\vec {p}_2$ to remove $(d-1)$ delta-functions

B.2 $$\begin{aligned} I= & {} \int [df_{1}][df_{3}][df_{4}]\frac{2\pi }{2E_2} \nonumber \\&\times \delta (m_{H}-E_1-E_2(\vec {p}_1,\vec {p}_3,\vec {p}_4)-E_3-E_4), \end{aligned}$$

with $E_2(\vec {p}_1,\vec {p}_3,\vec {p}_4) =|\vec {p}_{1}+\vec {p}_{3}+\vec {p}_{4}|$. We then integrate over $E_1$. It is difficult to use $\cos \theta _{24}$ as an independent variable, since $E_{2}$ is fixed by momentum conservation and thus $\cos \theta _{24}$ is a function of $E_2$ and of $E_1$. Instead, we parametrize the measure in terms $\{E_1,E_3,E_4,\vec {n}_1, \vec {n}_3,\cos \theta _{13,4}\}$ where $\vec {n}_i = \vec {p}_i/|\vec {p}_i|$ and $\theta _{13,4}$ is the angle between the vector $\vec {p}_{13} = | \vec {p}_1 + \vec {p}_3|$ and $\vec {p}_4$,

B.3 $$\begin{aligned} \cos \theta _{13,4}=\frac{\vec {p}_{13}}{|\vec {p}_{13}|} \cdot \vec {n}_4. \end{aligned}$$

The integral over $E_1$ removes the remaining delta function

B.4 $$\begin{aligned}&\int \mathrm{d}E_1 \delta (m_{H}-E_1-E_2(\vec {p}_1, \vec {p}_3,\vec {p}_4)-E_3-E_4) \nonumber \\&\quad \equiv \frac{1}{1+\frac{\partial E_2}{\partial E_1}}, \end{aligned}$$

where now all values of $E_1$ should be evaluated at $E_1=E_1^*$ which fulfils the $\delta$-function constraint in the above equation. We obtain

B.5 $$\begin{aligned} I = \int [df_{3}] [df_{4}]\frac{\mathrm{d}\vec {\Omega }_1}{4(2\pi )^{d-2}} E_1^{-2\epsilon } \left[ \frac{E_1}{E_2}\frac{1}{1+\frac{\partial E_2}{\partial E_1}} \right] . \end{aligned}$$

We now compute $\frac{\partial E_1}{\partial E_2}$. We use

B.6 $$\begin{aligned} E_2^2= & {} |\vec {p}_{13}+\vec {p}_4|^2 \nonumber \\= & {} |p_{13}|^2+E_4^2+2E_4 |\vec {p}_{13}| \cos \theta _{13,4}, \end{aligned}$$

and $|\vec {p}_{13}|^2=E_1^2+E_3^2+2E_1E_3\cos \theta _{13}$ to get

B.7 $$\begin{aligned} \frac{\partial E_2}{\partial E_1} = \frac{E_1+E_3\cos \theta _{13}}{E_2} \left[ 1+\frac{E_4}{|\vec {p}_{13}|} \cos \theta _{13,4}\right] . \end{aligned}$$

We can also rewrite the angle between vectors $\vec {p}_{13}$ and $\vec {p}_4$ through the angle between $\vec {p}_2$ and $\vec {p}_4$. Indeed using

B.8 $$\begin{aligned} \cos \theta _{13,4}= & {} \vec {n}_{13} \cdot \vec {n}_4 = \frac{\vec {p}_{13}}{|\vec {p}_{13}|} \cdot \vec {n}_4 \nonumber \\= & {} -\frac{\vec {p}_{24}}{|\vec {p}_{13}|} \cdot \vec {n}_4=-\frac{E_4+E_2 \cos \theta _{24}}{|\vec {p}_{13}|}, \end{aligned}$$

in Eq. (B.7), we find

B.9 $$\begin{aligned} \frac{\partial E_2}{\partial E_1} = \frac{E_1+E_3\cos \theta _{13}}{E_2} \left[ 1-\frac{E_4^2 +E_2 E_4 \cos \theta _{24}}{|\vec {p}_{13}|^2} \right] . \end{aligned}$$

Finally, we use $|\vec {p}_{13}|^2=|\vec {p}_{24}|^2 =E_2^2+E_4^2+2E_2E_4\cos \theta _{24}$ and obtain

B.10 $$\begin{aligned} \frac{\partial E_2}{\partial E_1} = \frac{E_1+E_3\cos \theta _{13}}{|\vec {p}_{13}|^2} \left[ E_2+ E_4 \cos \theta _{24}\right] . \end{aligned}$$

We now compute the energies $E_1^*$ and $E_2$ that are supposed to be used in all the formulas. Squaring both sides of the equation $p_H-p_1-p_3=p_2+p_4$, we obtain

B.11 $$\begin{aligned}&m_{H}^2 -2m_{H}(E_1+E_3)+2E_1 E_3(1-\cos \theta _{13}) \nonumber \\&\quad = 2E_2 E_4(1-\cos \theta _{24}). \end{aligned}$$

We further use the energy conservation equation $E_2 =m_{H}-E_1-E_3-E_4$ to find

B.12 $$\begin{aligned} E_1^*=\frac{m_{H}^2-2m_{H}E_3 - 2(m_{H}-E_3-E_4)E_4 (1-\cos \theta _{24})}{2[m_{H}-E_3(1-\cos \theta _{13})-E_4(1-\cos \theta _{24})]}. \end{aligned}$$

The energy $E_2$ is then obtained from energy conservation.

Finally, we write the phase space for parton $f_4$ in terms of the angle $\theta _{13,4}$

B.13 $$\begin{aligned}{}[df_{4}] = \frac{\mathrm{d}E_4 E_4^{1-2\epsilon }}{2(2\pi )^{d-1}} \mathrm{d}\cos \theta _{13,4} (1-\cos ^2\theta _{13,4})^{-\epsilon }\mathrm{d}\Omega _{13,4}, \end{aligned}$$

To rewrite it in terms of $\theta _{24}$, we use Eq. (B.8)

B.14 $$\begin{aligned} 1-\cos ^2\theta _{13,4}= & {} 1 -\frac{(E_4+E_2 \cos \theta _{24})^2}{|\vec {p}_{13}|^2}\nonumber \\= & {} \frac{E_2^2}{|\vec {p}_{13}|^2} \left( 1-\cos ^2\theta _{24}\right) , \end{aligned}$$

where we applied the equality $\vec {p}_{13}=-\vec {p}_{24}$. The Jacobian that originates from the variable change $\cos \theta _{13,4} \rightarrow \cos \theta _{24}$ is computed employing Eq. (B.8) one more time. The result reads

B.15 $$\begin{aligned} J_{\Omega }= & {} \frac{\partial \cos \theta _{13,4}}{\partial \cos \theta _{24}}\nonumber \\= & {} -\left[ \frac{E_2}{|\vec {p}_{13}|}+ \cos \theta _{24} \frac{\partial E_2}{\partial \cos \theta _{24}}\frac{1}{|\vec {p}_{13}|}\right. \nonumber \\&\left. - \frac{E_4+E_2\cos \theta _{24}}{|\vec {p}_{13}|^2} \frac{\partial |\vec {p}_{13}|}{\partial \cos \theta _{24}}\right] . \end{aligned}$$

To simplify it, we use

B.16 $$\begin{aligned} \frac{\partial |\vec {p}_{13}|}{\partial \cos \theta _{24}}= & {} \frac{\partial |\vec {p}_{13}|}{\partial E_1} \frac{\partial E_1}{\partial \cos \theta _{24}} \nonumber \\= & {} \frac{E_1+E_3 \cos \theta _{13}}{|\vec {p}_{13}|} \frac{\partial E_1}{\partial \cos \theta _{24}}. \end{aligned}$$

Next we employ energy conservation to write $\partial E_2/\partial \cos \theta _{24}=-\partial E_1/\partial \cos \theta _{24}$, and applying $\partial /\partial \cos \theta _{24}$ to both sides of Eq. (B.11), we obtain

B.17 $$\begin{aligned} \frac{\partial E_1}{\partial \cos \theta _{24}}= & {} -\frac{\partial E_2}{\partial \cos \theta _{24}}\nonumber \\= & {} \frac{E_2 E_4}{m_{H}-E_3(1-\cos \theta _{13})-E_4(1-\cos \theta _{24})}.\nonumber \\ \end{aligned}$$

We use this result to write the Jacobian as

B.18 $$\begin{aligned} J_{\Omega }&=- \frac{E_2}{|\vec {p}_{13}|}\nonumber \\&\quad \times \Biggl \{1-\frac{E_4}{m_{H}-E_3(1-\cos \theta _{13})-E_4(1-\cos \theta _{24})}\nonumber \\&\quad \times \left( \cos \theta _{24} +\frac{(E_4+E_2\cos \theta _{24}) (E_1+E_3 \cos \theta _{13})}{|\vec {p}_{13}|^2} \right) \Biggr \}. \end{aligned}$$

Finally, applying

B.19 $$\begin{aligned} \frac{E_4^2+E_2E_4\cos \theta _{24}}{|\vec {p}_{13}|^2} =1-\frac{E_2^2+E_2E_4\cos \theta _{24}}{|\vec {p}_{13}|^2}, \end{aligned}$$

we obtain the final formula for the Jacobian

B.20 $$\begin{aligned} J_{\Omega }= & {} -\frac{E_2^2}{|\vec {p}_{13}|}\nonumber \\&\times \frac{1}{m_{H}-E_3(1-\cos \theta _{13}) -E_4( 1-\cos \theta _{24})}\nonumber \\&\times \Biggl [1+\frac{(E_2+E_4\cos \theta _{24})(E_1+E_3 \cos \theta _{13})}{|\vec {p}_{13}|^2}\Biggr ].\nonumber \\ \end{aligned}$$

The non-trivial factor present in Eq. (B.5) multiplied with the Jacobian in Eq. (B.20) simplifies to

B.21 $$\begin{aligned}&\left| \frac{E_1}{E_2} J_{\Omega } \frac{1}{1+\frac{\partial E_2}{\partial E_1}}\right| \nonumber \\&\quad =\frac{E_1 E_2}{|\vec {p}_{13}|\big [m_{H}-E_3 ( 1- \cos \theta _{13}) -E_4(1-\cos \theta _{24}) \big ]}.\nonumber \\ \end{aligned}$$

We employ this result to derive our final formula for the phase-space integral that we use to describe double-collinear contributions

B.22 $$\begin{aligned} I= & {} \int [df_{3}] \frac{\mathrm{d}E_4 E_4^{1-2\epsilon }}{2(2\pi )^{d-1}} \mathrm{d}\cos \theta _{24}(1-\cos ^2\theta _{24})^{-\epsilon } \nonumber \\&\times \left[ \frac{E_1^2 E_2^2}{|\vec {p}_{13}|^2}\right] ^{-\epsilon } \frac{\mathrm{d}\Omega _1\mathrm{d}\Omega _{13,4}}{4(2\pi )^{d-2}} \nonumber \\&\times \frac{E_1 E_2}{|\vec {p}_{13}|\big [m_{H}-E_3 ( 1- \cos \theta _{13}) -E_4(1-\cos \theta _{24}) \big ]}.\nonumber \\ \end{aligned}$$

The phase space for $[df_{3}]$ is generated using the relative angle between $\vec {p}_1$ and $\vec {p}_3$ as a variable.

## Appendix C: Prompt decays of the Higgs boson to $bb\bar{b} \bar{b}$ final states

In this appendix, we consider the prompt decay of the Higgs boson^13^ to four *b*-quarks

C.1 $$\begin{aligned} H \rightarrow b_A + \bar{b}_B + b_C + \bar{b}_D. \end{aligned}$$

There are four subamplitudes that contribute to this process; they are shown in Fig. 1. The difference between these amplitudes is in the fermion lines that originate from the $Hb \bar{b}$ vertex and the ones that originate from the gluon splitting, $g^* \rightarrow b \bar{b}$. It is clear that *b*-quarks from the $H \bar{b} b$ vertex are hard, in a sense that they cannot produce infra-red singularities, whereas *b*-quarks from gluon splitting can be soft. Since whether a given *b*-quark is hard or soft changes from diagram to diagram, the extraction of singularities becomes intricate.

To overcome this problem we make use of the symmetries of the $H\rightarrow bb\bar{b}\bar{b}$ decay. To this end, we write the matrix element as the sum of four subamplitudes shown in Fig. 1

C.2 $$\begin{aligned} \mathcal {M} = m_a + m_b + m_c + m_d \end{aligned}$$

and square it. Introducing the notation $m_{ij}=2\mathrm{Re}(m_i m_j^*)$ to describe interferences of subamplitudes, we obtain

C.3 $$\begin{aligned} |\mathcal {M}|^2= & {} \sum _{i=a,\ldots ,d} |m_i|^2 + m_{ab} + m_{ac} \nonumber \\&+ m_{ad} + m_{bc} + m_{bd} + m_{cd}. \end{aligned}$$

The $H \rightarrow b\bar{b}b\bar{b}$ decay width reads

C.4

In the squares of amplitudes, the choice of (potentially) hard and soft fermions is unambiguous. We label the hard momenta as 1 and 2 and the soft momenta as 3 and 4. Using the symmetry of the phase space, we obtain

C.5

where we have included a factor of 4 for the four diagrams and another factor of 2 for the energy ordering $E_3>E_4$. It is straightforward to extract the various singularities from this contribution; in fact the result is identical to the $q \bar{q}$ contribution to NNLO QCD corrections to $H \rightarrow b \bar{b}$ decay.

The interference terms in Eq. (C.4) are more involved since it is not possible to choose hard and soft momenta unambiguously. Before discussing this, we note that since helicities of massless quarks are conserved and since $H \rightarrow b \bar{b}$ and $g^* \rightarrow b \bar{b}$ produce quarks with different (same) helicities, respectively, the interferences of diagrams (*a*) and (*d*) $m_{ad}$ as well as diagrams (*b*) and (*c*) $m_{bc}$ vanish. We then classify the possible collinear divergences in the remaining interference contributions. We find the following divergences in various interference terms:

- there is a triple-collinear singularity in $m_{ab}$ when $f_B,f_C$ and $f_D$ are collinear;
- there is a triple-collinear singularity in $m_{ac}$ when $f_A,f_C$ and $f_D$ are collinear;
- there is a triple-collinear singularity in $m_{bd}$ when $f_A,f_B$ and $f_C$ are collinear;
- there is a triple-collinear singularity in $m_{cd}$ when $f_A,f_B$ and $f_D$ are collinear.

Out of these four interferences, only two are independent. The relations are

C.6 $$\begin{aligned}&m_{bd}(A_b,B_{\bar{b}},C_b,D_{\bar{b}}) = m_{ac}(A_b,D_{\bar{b}},C_b,B_{\bar{b}}),\nonumber \\&m_{cd}(A_b,B_{\bar{b}},C_b,D_{\bar{b}}) = m_{ab}(C_b,B_{\bar{b}},A_b,D_{\bar{b}}). \end{aligned}$$

We also note that

C.7 $$\begin{aligned}&m_{ab}(A_b,B_{\bar{b}},C_b,D_{\bar{b}} ) = m_{ab}(A_b,D_{\bar{b}},C_b,B_{\bar{b}}),\nonumber \\&m_{ac}(A_b,B_{\bar{b}},C_b,D_{\bar{b}}) = m_{ac}(C_b,B_{\bar{b}},A_b,D_{\bar{b}}). \end{aligned}$$

Using these results, we can write

C.8

By construction, c.f. Fig. 2, the interference contributions are only singular in the limit when momenta of partons $f_1,f_3$ and $f_4$ become collinear, whereas the non-interference term has multiple singularities, including the double-soft one that occurs when $f_3$ and $f_4$ become soft. We denote the interference term as

C.9 $$\begin{aligned}&\langle F_\mathrm{LM}^\mathrm{int}(1,2,3,4)\rangle _{\delta } \nonumber \\&\quad = \langle \theta (E_3- E_4) \{ m_{ab}(2_b,3_{\bar{b}}, 1_b,4_{\bar{b}}) + m_{ac}(3_b,2_{\bar{b}},4_{b},1_{\bar{b}}) \} \rangle _\delta , \end{aligned}$$

and use this notation in the main text when we discuss the computation of NNLO QCD contribution to Higgs decay to two quarks in Sect. 5. The non-interference term in Eq. (C.8) is accounted for as part of the $n_f$-dependent contributions in the NNLO QCD computation.

## Footnotes

1 We note that in this paper we always work with UV-renormalized amplitudes.

2 In this section and in Sect. 4, we assume that the Higgs directly couples to gluons through an effective vertex.

3 We remind the reader that throughout this paper we will use $E_\mathrm{max}= m_{H}/2$.

4 Note that this choice is always well-defined because the configuration $p_1||p_2||p_3$ is kinematically not allowed.

5 The extra factor of 2 comes from a mismatch between the symmetry factors of $\left\langle F_\mathrm{LM}(1_q,2_g,3_{\bar{q}}) \right\rangle$ and $\left\langle F_\mathrm{LM}(1_g,2_g) \right\rangle$.

6 In this subsection, the “tree” superscript on $\mathcal {M}$ is always assumed.

7 We have used the $1\leftrightarrow 2$ symmetry to obtain this formula.

8 We emphasize that in this section, the Higgs boson does not couple to gluons but only to *b*-quarks. Furthermore, we assume that all quarks are massless, despite the *b*-quark having a non-vanishing Yukawa coupling.

9 To avoid confusion, we emphasize that in the previous section a 4*q* final state originating from the decay $H \rightarrow (g^*\rightarrow q \bar{q}) \; (g^* \rightarrow q \bar{q})$ was discussed whereas in this section we consider prompt decays to fermions. For this reason, the 4*b* final state originates from e.g. $H \rightarrow (b^* \rightarrow b \bar{b} b ) \; \bar{b}$ etc.

10 For our implementation, we take all the non-trivial amplitudes from Refs. [40, 41].

11 We note that similar calculations have been discussed earlier [45–62], see e.g. [63, 64] for an exhaustive list of references.
